# SNH Amidation of 5-Nitroisoquinoline: Access to Nitro- and Nitroso Derivatives of Amides and Ureas on the Basis of Isoquinoline

**DOI:** 10.3390/molecules27227862

**Published:** 2022-11-14

**Authors:** Elena K. Avakyan, Anastasia A. Borovleva, Diana Yu. Pobedinskaya, Oleg P. Demidov, Artem P. Ermolenko, Alexander N. Larin, Ivan V. Borovlev

**Affiliations:** Department of Chemistry, North Caucasus Federal University, 1a Pushkin St., 355017 Stavropol, Russia

**Keywords:** heterocycles, S_N_^H^ methodology, metal-free C–N bond formation, 5-nitroisoquinoline, redox process, oxidative S_N_^H^ amidation, S_N_^H^ dialkylcarbamoylamination, regioselectivity

## Abstract

For the first time, amides and ureas based on both 5-nitroisoquinoline and 5-nitrosoisoquinoline were obtained by direct nucleophilic substitution of hydrogen in the 5-nitroisoquinoline molecule. In the case of urea and monosubstituted ureas, only 5-nitrosoisoquinoline-6-amine is formed under anhydrous conditions.

## 1. Introduction

The molecular framework of isoquinoline is the basis of an extensive family of alkaloids exhibiting diverse biological activity [1,2,3], and their synthetic derivatives are widely used in pharmacology and medicine [4]. Thus, further functionalization of isoquinoline seems to be a very promising direction. Without detracting from the advantages of multistage functionalization methods, where the final stage is cyclization with the formation of an isoquinoline ring [5,6,7,8,9,10,11,12,13], we note that modern requirements for the synthesis of derivatives of aromatic and heteroaromatic compounds imply their direct C–H functionalization, the economy of all transformation parameters, which is in line with the so-called PASE (Pot-, Atom-, and Step-Economic) concept [14], as well as the principles of green chemistry [15].

In the case of π-deficient azines and nitroarenes, oxidative nucleophilic hydrogen substitution (S_N_^H^) reactions satisfy these requirements, which do not require the preliminary introduction of a good leaving group into the substrate or reagent molecule, as well as the use of expensive catalysts and ligands [16,17,18,19]. They include an additional step with the formation of σ^H^ adduct and its subsequent aromatization due to an external oxidizing agent. Organic and inorganic compounds and atmospheric oxygen are used to oxidize σ^H^ adducts [20,21], while electrochemical oxidation is used for stable intermediates [22,23,24]. Even in the absence of an external oxidant, the NO_2_ [25] group or the C=N bond of the substrate [26] can also act as hydride anion acceptors. The most probable mechanism for the dehydroaromatization of the σ^H^ complex is the successive transfer of an electron, a proton, and one more electron (EPE mechanism) to the oxidant molecule [27]. Later, in the arylamination of nitroarenes, in addition to the oxidative one, another way of aromatization of the σ^H^ adduct was discovered by its dehydration with the formation of the corresponding nitroso compounds [28,29,30,31,32,33,34]. On the whole, the S_N_^H^ methodology has already found application in industry [35,36] and, in some cases, is a good alternative to cross-coupling reactions with the participation of transition metals [37].

The aim of the first stage of the work was to study the possibility of direct nucleophilic substitution of hydrogen by the N-amide function in the 5-nitroisoquinoline molecule. It is known that this compound readily enters into reactions of oxidative amination [38], methylamination [39], whereas its quinoline analogue even into arylamination reaction [40]. However, unlike other N-nucleophiles, reactions of direct hydrogen substitution by the N-amide function in the series of azines and nitroarenes are still quite rare. The first report on the S_N_^H^ amidation of nitrobenzene appeared only in 1993 [41] during the development of an industrial method for the preparation of *p*-nitroaniline [42].

In continuation of these studies, the oxidative S_N_^H^ amidation of 1,3,7-triazapyrene [43], acridine [44], 3-nitropyridine [45] and 5(6,7,8)-nitroquinolines [46] was successfully performed in our laboratory. In all cases, the process was carried out in anhydrous DMSO by the action on the substrate with the previously obtained anion of the corresponding carboxamide at room temperature, using atmospheric oxygen [43,46] or K_3_Fe(CN)_6_ [44,45] as an oxidizing agent.

## 2. Results and Discussion

**Scheme 1:**
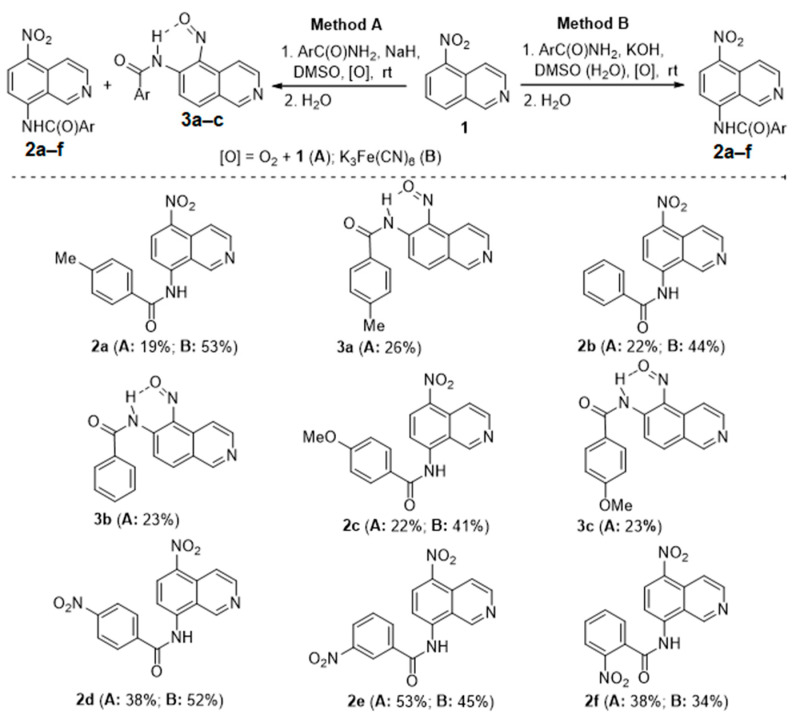
S_N_^H^ Amidation of 5-nitroisoquinoline.

We implemented two approaches to the S_N_^H^ amidation of 5-nitroisoquinoline (**1**), which differed mainly in the presence or absence of a small amount of water in the reaction mass, and which led to significantly different results (Scheme 1). The optimization of the first of them (method A) was carried out using the example of the reaction of substrate **1** with p-methylbenzamide in anhydrous DMSO at room temperature, preliminarily generating the amide anion by the action of NaH in the same solvent. The best result was shown by using 2 equiv. amide anion per 1 equiv. substrate (Table 1, entry 1). After adding 5-nitroisoquinoline (**1**), the process has completed within 1.5 h with the formation of a mixture of 4-methyl-*N*-(5-nitroisoquinolin-8-yl)benzamide (**2a**) and 4-methyl-*N*-(5-nitrosoisoquinoline-6-yl)benzamide (**3a**) with a total yield of 45%, the separation of which was carried out by chromatography on silica gel. All spectral data are available in the Supplementary Materials file submitted with this article.

Increasing the excess of the amide anion (entries 2,3), changing the ratio of *p*-methylbenzamide and NaH (entries 4,5), using K_3_Fe(CN)_6_ as an external oxidizer (entry 6), and increasing the temperature (entry 7) turned out to be ineffective. The reaction was carried out without isolation from air oxygen; however, its performance in an argon atmosphere only slightly reduced the yield of nitro product **2a** (entry 8). These data suggest that, as in the case of 3-nitropyridine [45] and 5-nitroquinoline [46], 5-nitroisoquinoline exhibits a dual reactivity, being not only a substrate, but also the main oxidizer of σ^H^ adducts in the formation of nitro-amides **2**. Naturally, this reduces the yield of target compounds and leads to the appearance of by-products.

Anions of benzamide- and p-methoxybenzamide react similarly with the formation of the corresponding nitroamides **2b,c** and nitrosoamides **3b,c**. Note, however, that the reaction of the initial substrate with *p*-, *m*-, and *o*-nitrobenzamides gives only nitroamides **2d**–**f** (Scheme 1).

**Scheme 2:**
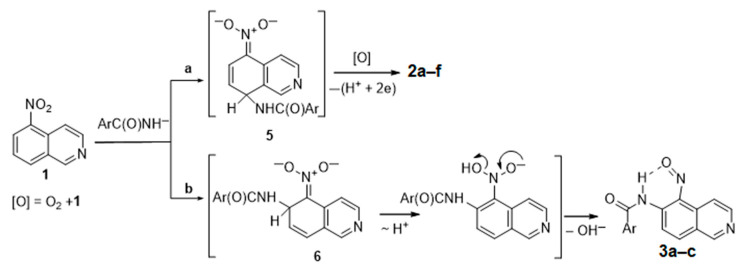
Proposed mechanism for the synthesis of nitro- **2a**–**f** and nitroso compounds **3a**–**c**.

The mechanism of amidation of 5-nitroisoquinoline (**1**) includes the addition of a nucleophile in the *ortho*- and *para*-positions towards the NO_2_-group, and the *para* σ^H^ adduct **5** further undergoes oxidative aromatization to form nitroamides **2a**–**f** (Scheme 2, route **a**), while its *ortho* analog **6** is aromatized by proton transfer and elimination of a water molecule, giving nitrosoamides **3a**–**c** (Scheme 2, route **b**).

The starting point for the development of another approach to the S_N_^H^ amidation of 5-nitroisoquinoline (**1**) was the fact that the authors of the first work on amidation by direct hydrogen substitution [41] carried out the process in a non-absolute medium, using dihydrate of tetramethylammonium hydroxide as the base, which led to the formation of only products substitution at the *p*-position of the nitrobenzene molecule. In this approach, we used commercial DMSO containing ~0.5% water as well as KOH instead of NaH as the base (method B). As it turned out, when using a 4-molar excess of the corresponding aromatic amides, only the corresponding N-(5-nitroisoquinolin-8-yl)benzamides **2a**–**f** were formed in 34–53% yields (Scheme 1). Apparently, in the presence of water, the hydrated amide anions, which have a large volume, experience steric difficulties in entering the *o*-position with respect to the NO_2_ group, i.e., in position 6 of the 5-nitroisoquinoline molecule. Despite relatively low yields, nitro- **2** and nitroso compounds **3** are of considerable interest for further functionalization of isoquinoline, and it is very problematic to obtain nitrosoamides **3a**–**c** by other methods.

Increasing the water content in the mixture with DMSO to 5% in method B, or using amides of aliphatic acids (acetic, propionic and isobutyric) under the conditions of both methods, led to a complex mixture of substances.

**Figure 1:**
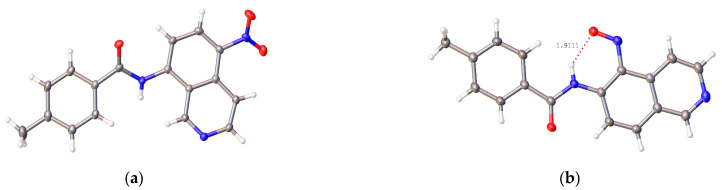
(**a**) ORTEP diagram of nitro compound **2a**; (**b**) ORTEP diagram of nitroso compound **3a** (The dashed line shows the intramolecular hydrogen bond). The crystallographic data could be found in the Supplementary Materials.

A feature of the ^1^H NMR spectra of nitrosoamides **3a**–**c** in CDCl_3_ is a strong downfield shift of NH proton signals (δ~13.5–13.6 ppm), which indicates a strong intramolecular hydrogen bond NH^…^O=N. The structures of the compounds **2a** (CCDC 2159575) and **3a** (CCDC 2159573) (Figure 1) were confirmed by X-ray determination [47].

The aim of the second stage of the work was to study the possibility of S_N_^H^ amidation of 5-nitroisoquinoline with urea and its derivatives. We have previously shown that unsubstituted urea can act as an aminating agent in nucleophilic substitution reactions [47,48,49,50]. For example, the reaction of acridine with the urea anion in anhydrous DMSO led to 9-aminoacridine in 78% yield [50]. However, alkylureas under the same conditions entered into the S_N_^H^ alkyl(dialkyl)carbamoylamination reaction, allowing to introduce the residues of the corresponding ureas into acridine [50] and 3-nitropyridine [51] molecules. Under anhydrous conditions, urea anions easily form stable ϭ-adducts at position 9 with 10-alkylacridinium cations [52].

**Scheme 3:**
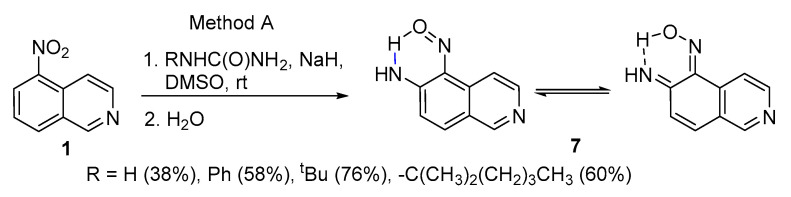
Synthesis and tautomerism of compound **7**.

We have found that urea and its monosubstituted derivatives such as phenyl-, tert-butyl- and (1,1-dimethylpentyl)urea react with 5-nitroisoquinoline under anhydrous conditions (method A) to form the same compound—5-nitrosoisoquinoline-6-amine (**7**; Scheme 3). In the ^1^H NMR spectrum of compound **7**, even in such a polar solvent as DMSO-d_6_, the NH_2_ group gives two broadened singlets at δ 11.53 and 8.90 ppm, the first of which corresponds to a proton bound by a strong intramolecular hydrogen bond NH^…^O= N. In the ^13^C NMR spectrum, the signals at δ 136.6 and 139.2 ppm appear only with increasing accumulation time and are strongly broadened. In our opinion, this is the result of the well-known prototropic tautomerism of the nitrosamine-azaquinone oxime type [53,54,55] (Scheme 3), and the tautomerization rate for this compound is relatively slow in the NMR time scale, and these signals refer to the C_5_ and C_6_ atoms of the isoquinoline cycle. All spectral data are available in the Supplementary Materials file submitted with this article.

**Scheme 4:**
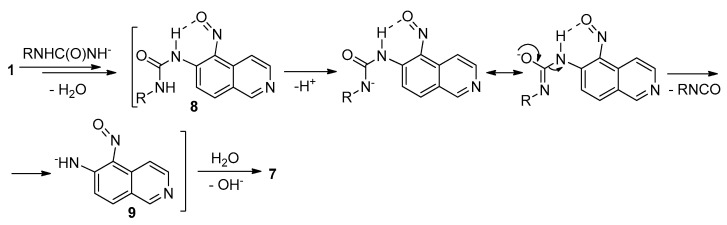
Proposed pathway for the formation of the compound **7**.

Unlike amides, S_N_^H^ reactions of 5-nitroisoquinoline with ureas under anhydrous conditions (method A) proceed exclusively at position 6 in accordance with the mechanism of formation of nitrosoamides **3a**–**c** (Scheme 2, route **b**). However, the nucleophilic substitution product **8** is unstable, and under the reaction conditions, the urea radical is converted into an amino group according to the route we proposed earlier [43,48] (Scheme 4). The key to it is the elimination of the isocyanic acid molecule or its ester (RNCO) and the formation of the anion **9**.

Application of the conditions of method B to the reaction of 5-nitroisoquinoline with anions of urea and its monosubstituted derivatives was unsuccessful, since a complex mixture of substances is formed in the presence of water.

**Scheme 5:**
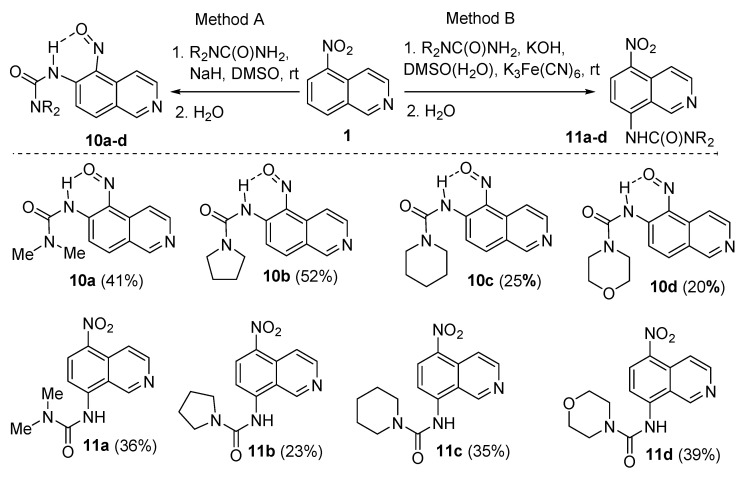
S_N_^H^ Reactions of 5-nitroisoquinoline (**1**) with an ureas.

However, 1,1-dialkylurea anions react with 5-nitroisoquinoline (**1**) under the conditions of both methods, but form different substitution products, albeit in relatively low yields (Scheme 5). So, under anhydrous conditions (method A), the 1,1-dimethylurea anion leads to the S_N_^H^ product at position 6—1,1-dimethyl-3-(5-nitrosoisoquinolin-6-yl)urea (**10a**) in 41% yield. Amides of pyrrolidine-1-carboxylic, piperidine-1-carboxylic, and morpholine-4-carboxylic acids also react similarly, forming ureas based on 5-nitrosoisoquinoline **10b**–**d**. Undoubtedly, the mechanism of formation of compounds **10a**–**d** corresponds to the general mechanism for obtaining nitroso compounds (Scheme 2, route **b**).

In the ^1^H NMR spectra of these compounds, the NH proton signal is strongly shifted downfield in both CDCl_3_ and DMSO-d_6_ (δ~13.5–13.6 ppm), which also indicates a strong intramolecular hydrogen bond. In addition, if under normal conditions of recording the spectrum (25 °C) in CDCl_3_, the protons of two methyl groups of compound **10a** give one broadened singlet, then when cooled to only 13.7 °C, it splits into two signals, which indicates the nonequivalence of methyl groups under these temperature conditions. In our opinion, this is due to the well-known difficulty in rotation relative to the C(O)-N amide bond. At 25 °C the rotation accelerates and the signals from these groups coalesce. However, in the case of N-(5-nitrosoisoquinolin-6-yl)pyrrolidine-1-carboxamide (**10b**), the protons of both the α- and β-methylene groups of the pyrrolidine ring are not equivalent even at 25 °C and give separate signals (as do the signals of carbon atoms of these groups in ^13^C NMR). The difficulty of rotation relative to the amide bond of compounds **10c** and **10d** is noticeable only in the broadening of the signals of the corresponding protons, which appear equivalent. Apparently, this is due to the lower conformational rigidity of the piperidine and morpholine rings of compounds **10c** and **10d** compared to the pyrrolidine one.

**Figure 2:**
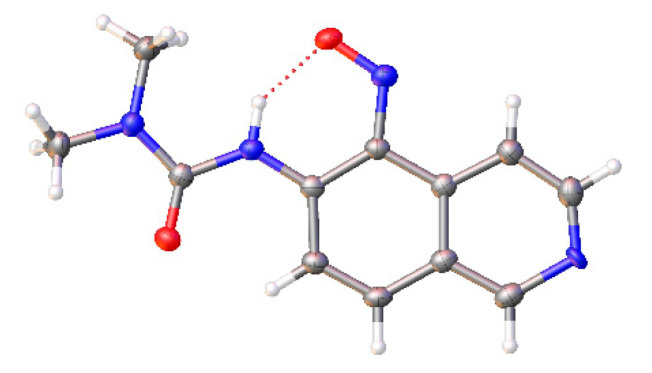
ORTEP diagram of nitroso compound **10a.** The crystallographic data could be found in the Supplementary Materials.

In the presence of water (method B), the reactions of 5-nitroisoquinoline with 1,1-dialkylurea anions proceed by the mechanism of oxidative nucleophilic substitution (Scheme 2, route **a**) to position 8 with the formation of exclusively S_N_^H^ dialkylcarbamoylamination products **11a**–**d** (Scheme 5). The structure of the compound **10a** (CCDC 2159579, Figure 2) was confirmed by X-ray determination [47].

## 3. Materials and Methods

^1^H and ^13^C NMR spectra were recorded on a Bruker Avance HD 400 spectrometer in the solvent indicated relative to residual DMSO signals [56] or TMS as internal standard when CDCl_3_ was used as a solvent. ard—85% H3PO4. The NMR-spectra of the newly synthesized compounds could be found in the Supplementary Materials. HRMS were registered on a Bruker UHR-TOF Maxis™ Impact instrument using the ESI technique. All melting points were determined in glass capillaries using REACH Devices RD-MP and Electrothermal IA 9200 instruments and are uncorrected. The reaction progress and the purity of the obtained compounds were controlled by TLC on Silufol UV-254 plates. All experiments were carried out in a reactor protected from atmospheric moisture, but without isolation from atmospheric oxygen. Sodium hydride (60% paraffin oil suspension, Merck, Darmstadt, Germany) and 5-nitroisoquinoline (abcr GmbH & Co. KG, Heilbronn, Germany) were used without further purification.

*N-(5-nitroisoquinolin-8-yl)benzamides* **2a**–**f** and *N-(5-nitrosoisoquinoline-6-yl)benzamides* **3a**–**c**; *(General Procedures):*

Method A: To a solution of 1 mmol of the corresponding benzamide in 4 mL of anhydrous DMSO 40 mg of a suspension of sodium hydride in paraffin oil (1 mmol of NaH) and after 10 min 87 mg (0.5 mmol) 5-nitroisoquinoline were added sequentially at room temperature. The mixture was intensively stirred at room temperature for 1.5 h. Then, the reaction mass was poured into 50 g of ice and, upon reaching room temperature, it was acidified with dilute HCl to pH~7. The precipitate that formed was filtered off, washed with water, and dried. The dry product was separated into the appropriate fractions by dry flash chromatography [57] on silica gel. In the synthesis of compounds **2a**–**c** and **3a**–**c**, the mixture was eluted with toluene—ethyl acetate (15:1) and the second and third fractions were collected (the first one, slightly colored fraction was discarded; it contains the starting benzamides and non-polar impurities). Nitrosoamides **3a**–**c** were obtained from the second green fraction, and nitroamides **2a**–**c** were obtained from the third yellow fraction. In the synthesis of compounds **2d**–**f**, the mixture was eluted with toluene—ethyl acetate (5:1) and the second yellow fraction was collected.

Method B: To a solution of 2 mmol of the corresponding benzamide in 8 mL of DMSO containing 0.5% water, 112 mg of KOH (2 mmol), 87 mg (0.5 mmol) of 5-nitroisoquinoline, and 658 mg (2 mmol) of K_3_Fe(CN)_6_ were added sequentially at room temperature. The mixture was vigorously stirred at room temperature for 2.5 h, then the reaction mixture was poured into 50 g of ice and, upon reaching room temperature, acidified with dilute HCl to pH~7. The precipitate was filtered off, washed with water, and dried. The dry product was purified by dry flash chromatography on silica gel eluting with benzene-ethyl acetate (5:1) and collecting a second yellow fraction.

*4-Methyl-N-(5-nitroisoquinolin-8-yl)benzamide* (**2a**). Yellow solid; yield: 29.2 mg (19%, Method A); 81.4 mg (53%, Method B); mp 205–206 °C (EtOAc). ^1^H NMR (400 MHz, DMSO-d_6_): δ = 11.01 (br s, 1H, NH), 9.68 (d, *J* = 0.5 Hz, 1H, H-1), 8.79 (d, *J* = 6.2 Hz, 1H, H-3), 8.74 (d, *J* = 8.6 Hz, 1H, H-6), 8.45 (d, *J* = 6.2 Hz, 1H, H-4), 8.15 (d, *J* = 8.6 Hz, 1H, H-7), 8.04 (d, *J* = 8.1 Hz, 2H, H-2,6 Ar), 7.42 (d, *J* = 8.1 Hz, 2H, H-3,5 Ar), 2.43 (s, 3H, CH_3_). ^13^C NMR (100 MHz, DMSO-*d*_6_): δ = 166.6, 149.4, 146.3, 142.6, 141.9, 140.4, 130.9, 129.9, 129.1, 128.5, 128.3, 122.2, 121.2, 115.1, 21.1. HRMS (ESI): *m/z* [M + Na]^+^ calcd for C_17_H_13_N_3_NaO_3_: 330.0849; found: 330.0831.

*4-Methyl-N-(5-nitrosoisoquinolin-6-yl)benzamide* (**3a**). Green solid; yield: 37.8 mg (26%, Method A); mp 168–169 °C (dec., EtOAc). ^1^H NMR (400 MHz, CDCl_3_): δ = 13.57 (br s, 1H, NH^…^O), 9.76 (d, *J* = 6.6 Hz, 1H, H-4), 9.73 (s, 1H, H-1), 9.56 (d, *J* = 9.5 Hz, 1H, H-8), 8.84 (d, *J* = 6.6 Hz, 1H, H-3), 8.64 (d, *J* = 9.5 Hz, 1H, H-7), 8.11 (d, *J* = 8.1 Hz, 2H, H-2,6 Ar), 7.50 (d, *J* = 856.1 Hz, 2H, H-3,5 Ar), 2.53 (s, 3H, CH_3_). ^13^C NMR (100 MHz, CDCl_3_): δ = 168.2, 146.1, 145.7, 145.4, 142.3, 141.1, 136.4, 130.4, 129.5, 128.6, 127.9, 124.3, 123.1, 119.3, 22.0. HRMS (ESI): *m/z* [M + H]^+^ calcd for C_17_H_14_N_3_O_2_: 292.1081; found: 292.1072.

*N-(5-Nitroisoquinolin-8-yl)benzamide* (**2b**)**.** Yellow solid; yield: 32.2 mg (22%, Method A); 64.5 mg (44%, Method B); mp 221–222 °C (EtOAc). ^1^H NMR (400 MHz, DMSO-d_6_): δ = 11.09 (br s, 1H, NH), 9.70 (s, 1H, H-1), 8.80 (d, *J* = 6.1 Hz, 1H, H-3), 8.75 (d, *J* = 8.6 Hz, 1H, H-6), 8.45 (d, *J* = 6.1 Hz, 1H, H-4), 8.17 (d, *J* = 8.6 Hz, 1H, H-7), 8.13 (d, *J* = 8.1 Hz, 2H, H-2,6 Ph), 7.69 (t, *J* = 7.7 Hz, 1H, H-4 Ph), 7.64–7.59 (m, 2H, H-3,5 Ph). ^13^C NMR (100 MHz, DMSO-*d*_6_): δ = 166.8, 149.4, 146.3, 141.8, 140.5, 133.8, 132.4, 129.8, 128.6, 128.5, 128.3, 122.2, 121.3, 115.1. HRMS (ESI): *m/z* [M + Na]^+^ calcd for C_16_H_11_N_3_NaO_3_: 316.0693; found: 316.0692.

*N-(5-Nitrosoisoquinolin-6-yl)benzamide* (**3b**). Green solid; yield: 31.9 mg (23%, Method A); mp 173–174 °C (dec., EtOAc). ^1^H NMR (400 MHz, CDCl_3_): δ = 13.53 (br s, 1H, NH^…^O), 9.41 (br s, 1H, H-1), 9.36–9.33 (m, 2H, H-4,8), 8.88 (d, *J* = 6.1 Hz, 1H, H-3), 8.43 (d, *J* = 9.4 Hz, 1H, H-7), 8.20 (d, *J* = 7.0 Hz, 2H, H-2,6 Ph), 7.74–7.65 (m, 3H, H-3,4,5 Ph). ^13^C NMR (100 MHz, CDCl_3_): δ = 168.5, 150.6, 147.6, 145.7, 141.5 (2C), 133.9 (2C), 133.0, 129.6, 128.4, 124.3, 121.0, 116.4. HRMS (ESI): *m/z* [M + H]^+^ calcd for C_16_H_12_N_3_O_2_: 278.0924; found: 278.0919.

*4-Methoxy-N-(5-nitroisoquinolin-8-yl)benzamide* (**2c**). Yellow solid; yield: 35.5 mg (22%, Method A); 66.2 mg (41%, Method B); mp 214–215 °C (EtOAc). ^1^H NMR (400 MHz, DMSO-d_6_): δ = 10.93 (br s, 1H, NH), 9.67 (s, 1H, H-1), 8.79 (d, *J* = 6.3 Hz, 1H, H-3), 8.73 (d, *J* = 8.6 Hz, 1H, H-6), 8.45 (d, *J* = 6.3 Hz, 1H, H-4), 8.14 (d, *J* = 8.6 Hz, 1H, H-7), 8.12 (d, *J* = 8.9 Hz, 2H, H-2,6 Ar), 7.14 (d, *J* = 8.9 Hz, 2H, H-3,5 Ar), 3.88 (s, 3H, CH_3_O). ^13^C NMR (100 MHz, DMSO-*d*_6_): δ = 166.0, 162.6, 149.5, 146.3, 142.1, 140.3, 130.3, 129.9, 128.5, 125.8, 122.1, 121.1, 115.1, 113.8, 55.6. HRMS (ESI): *m/z* [M + Na]^+^ calcd for C_17_H_13_N_3_NaO_4_: 346.0798; found: 346.0798.

*4-Methoxy-N-(5-nitrosoisoquinolin-6-yl)benzamide* (**3c**). Green solid; yield: 35.3 mg (23%, Method A); mp 186–187 °C (dec., EtOAc). ^1^H NMR (400 MHz, CDCl_3_): δ = 13.64 (br s, 1H, NH^…^O), 9.31 (s, 1H, H-1), 9.29–9.24 (m, 2H, H-4,8), 8.88 (d, *J* = 6.0 Hz, 1H, H-3), 8.34 (d, *J* = 9.4 Hz, 1H, H-7), 8.18 (d, *J* = 8.8 Hz, 2H, H-2,6 Ar), 7.14 (d, *J* = 8.8 Hz, 2H, H-3,5 Ar), 3.96 (s, 3H, °CH_3_). ^13^C NMR (100 MHz, CDCl_3_): δ = 168.0, 164.1, 151.9, 148.1, 148.0, 141.5, 130.6 (2C), 125.3, 124.4, 120.2, 115.5, 114.7 (2C), 55.8. HRMS (ESI): *m/z* [M + H]^+^ calcd for C_17_H_14_N_3_O_3_: 308.1030; found: 308.1030.

*4-Nitro-N-(5-nitroisoquinolin-8-yl)benzamide* (**2d**). Yellow solid; yield: 64.2 мг (38%, Method A); 87.9 mg (52%, Method B); mp 244–245 °C (EtOAc). ^1^H NMR (400 MHz, DMSO-d_6_): δ = 11.37 (br s, 1H, NH), 9.75 (s, 1H, H-1), 8.81 (d, *J* = 6.1 Hz, 1H, H-3), 8.76 (d, *J* = 8.6 Hz, 1H, H-6), 8.46-8.41 (m, 3H, H-4, H-2,6 Ar), 8.36 (d, *J* = 8.9 Hz, 2H, H-3,5 Ar), 8.18 (d, *J* = 8.6 Hz, 1H, H-7). ^13^C NMR (100 MHz, DMSO-*d*_6_): δ = 165.4, 149.6, 149.5, 146.4, 141.3, 140.8, 139.6, 129.9, 129.8, 128.5, 123.6, 122.3, 121.6, 115.2. HRMS (ESI): *m/z* [M + Na]^+^ calcd for C_16_H_10_N_4_NaO_5_: 361.0543; found: 361.0552.

*3-Nitro-N-(5-nitroisoquinolin-8-yl)benzamide* (**2e**). Yellow solid; yield: 89.6 mg (53%, Method A); 76.1 mg (45%, Method B); mp 215–216 °C (EtOAc). ^1^H NMR (400 MHz, DMSO-d_6_): δ = 11.38 (br s, 1H, NH), 9.74 (s, 1H, H-1), 8.95 (t, *J* = 1.8 Hz, 1H, H-2 Ar), 8.81 (d, *J* = 6.2 Hz, 1H, H-3), 8.76 (d, *J* = 8.6 Hz, 1H, H-6), 8.57–8.50 (m, 2H, H-4,6 Ar), 8.45 (d, *J* = 6.2 Hz, 1H, H-4), 8.15 (d, *J* = 8.6 Hz, 1H, H-7), 7.91 (t, *J* = 8.0 Hz, 1H, H-5 Ar). ^13^C NMR (100 MHz, DMSO-*d*_6_): δ = 164.8, 149.6, 147.8, 146.3, 141.2, 140.9, 135.3, 134.8, 130.3, 129.8, 128.5, 126.8, 123.1, 122.3, 121.8, 115.1. HRMS (ESI): *m/z* [M + Na]^+^ calcd for C_16_H_10_N_4_NaO_5_: 361.0543; found: 361.0546.

*2-Nitro-N-(5-nitroisoquinolin-8-yl)benzamide* (**2f**).Yellow solid; yield: 64.2 mg (38%, Method A); 57.5 mg (34%, Method B); mp 242–243 °C (EtOAc). ^1^H NMR (400 MHz, DMSO-d_6_): δ = 11.48 (br s, 1H, NH), 9.71 (s, 1H, H-1), 8.81 (d, *J* = 6.2 Hz, 1H, H-3), 8.79 (d, *J* = 8.4 Hz, 1H, H-6), 8.45 (d, *J* = 6.2 Hz, 1H, H-4), 8.27 (br d, *J* = 8.2 Hz, 2H, H-7, H-3 Ar), 8.03-7.95 (m, 2H, H-5,6 Ar), 7.87-7.81 (m, 1H, H-4 Ar). ^13^C NMR (100 MHz, DMSO-*d*_6_): δ = 165.8, 148.8, 146.5, 146.1, 140.8, 140.5, 134.6, 132.1, 131.4, 130.2, 129.6, 128.6, 124.4, 121.5, 120.1, 115.3. HRMS (ESI): *m/z* [M + Na]^+^ calcd for C_16_H_10_N_4_NaO_5_: 361.0543; found: 361.0524.

*5-Nitrosoisoquinolin-6-amine (**7**)*

To a solution of 1 mmol of the corresponding urea (Scheme 3) in 4 mL of anhydrous DMSO 40 mg of a suspension of sodium hydride in paraffin oil (1 mmol of NaH) and after 10 min 87 mg (0.5 mmol) 5-nitroisoquinoline were added sequentially at room temperature. The mixture was vigorously stirred at room temperature for 1 h. Then, the reaction mass was poured into 50 g of ice and, upon reaching room temperature, acidified with dilute HCl to pH~7. The precipitate formed was filtered off, washed with water, and dried. The dry product was purified by crystallization from ethyl acetate.

Green solid; yield: 32.9 mg (38%, from urea); 50.2 mg (58%, from phenylurea); 65.7 mg (76%, from *ter*t-butylurea); 51.9 mg (60%, from 1,1-dimethylpentylurea); mp 256–257 °C (dec., EtOAc). ^1^H NMR (400 MHz, DMSO-d_6_): δ = 11.53 (br s, 1H, NH^…^O), 9.03 (s, 1H, H-1), 8.90 (br s, 1H, NH), 8.82 (d, *J* = 5.7 Hz, 1H, H-4), 8.62 (d, *J* = 5.7 Hz, 1H, H-3), 8.02 (d, *J* = 9.3 Hz, 1H, H-8), 7.13 (d, *J* = 9.3 Hz, 1H, H-7). ^13^C NMR (100 MHz, DMSO-*d*_6_): δ = 151.2, 147.8, 147.7, 139.2, 137.9, 136.6, 122.1, 121.3, 112.9. HRMS (ESI): *m/z* [M + H]^+^ calcd for C_9_H_8_N_3_O: 174.0662; found: 174.0662.

*1,1-Dialkyl-3-(5-nitrosoisoquinolin-6-yl)ureas (**10a***–***d****); (General Procedure):*

To a solution of 1 mmol of the corresponding urea (Scheme 5, Method A) in 4 mL of anhydrous DMSO 40 mg of a suspension of sodium hydride in paraffin oil (1 mmol of NaH) and after 10 min 87 mg (0.5 mmol) 5-nitroisoquinoline were added sequentially at room temperature. The mixture was vigorously stirred at room temperature for 2 h. Then, the reaction mass was poured into 50 g of ice and, upon reaching room temperature, acidified with dilute HCl to pH~7. The precipitate formed was filtered off, washed with water, and dried. The dry product was isolated by dry flash chromatography^32^ on silica gel, collecting in all cases a second yellow or light green fraction, from which compounds **10a**–**d** were obtained. Eluents: PhMe- EtOAc (10:1) for **10a** and **10d**; PhMe- EtOAc (3:2) for **10b**; PhMe- EtOAc (1:1) for **10c**.

*1,1-Dimethyl-3-(5-nitrosoisoquinolin-6-yl)urea* (**10a**). Dark green solid; yield: 50.0 mg (41%); mp 161–162 °C (dec., PhMe-EtOAc). ^1^H NMR (400 MHz, DMSO-d_6_): δ = 13.43 (br s, 1H, NH^…^O), 9.30 (s, 1H, H-1), 8.96 (d, *J* = 5.8 Hz, 1H, H-3), 8.79 (d, *J* = 5.8 Hz, 1H, H-4), 8.77 (d, *J* = 9.4 Hz, 1H, H-8), 8.46 (d, *J* = 9.4 Hz, 1H, H-7), 3.14 (br s, 6H, N(CH_3_)_2_). ^1^H NMR (400 MHz, CDCl_3_, 25 ^°C^): δ = 13.69 (br s, 1H, NH^…^O), 9.23 (s, 1H, H-1), 9.17 (d, *J* = 5.8 Hz, 1H, H-3), 9.00 (d, *J* = 9.5 Hz, 1H, H-8), 8.81 (d, *J* = 5.8 Hz, 1H, H-4), 8.19 (d, *J* = 9.5 Hz, 1H, H-7), 3.26 (br s, 6H, N(CH_3_)_2_). ^1^H NMR (400 MHz, CDCl_3_, 13.7 ^°C^): δ = 13.58 (br s, 1H, NH^…^O), 9.56 (d, *J* = 6.3 Hz, 1H, H-3), 9.46 (s, 1H, H-1), 9.24 (d, *J* = 9.6 Hz, 1H, H-8), 8.79 (d, *J* = 6.3 Hz, 1H, H-4), 8.36 (d, *J* = 9.6 Hz, 1H, H-7), 3.45 (br s, 3H, N(CH_3_)**^b^**), 3.16 (br s, 3H, N(CH_3_)**^a^**). ^13^C NMR (100 MHz, CDCl_3_, 25 °C): δ = 154.8, 151.0, 148.1, 146.5, 141.0, 139.4, 129.0, 123.4, 120.3, 115.4, 37.0. HRMS (ESI): *m/z* [M + H]^+^ calcd for C_12_H_13_N_4_O_2_: 245.1033; found: 245.1030.

*N-(5-Nitrosoisoquinolin-6-yl)pyrrolidine-1-carboxamide* (**10b**). Green solid; yield: 70.2 mg (52%); mp 179–180 °C (dec., PhMe-EtOAc). ^1^H NMR (400 MHz, CDCl_3_, 25 °C): δ = 13.52 (br s, 1H, NH^…^O), 9.28 (br s, 1H, H-1), 9.26 (d, *J* = 6.2 Hz, 1H, H-3), 9.15 (br d, *J* = 9.5 Hz, 1H, H-8), 8.80 (d, *J* = 6.2 Hz, 1H, H-4), 8.22 (d, *J* = 9.5 Hz, 1H, H-7), 3.87 (t, *J* = 5.3 Hz, 2H, NCH_2_**^b^**), 3.59 (t, *J* = 5.3 Hz, 2H, NCH_2_**^a^**), 2.23–2.20 (m, 2H CH_2_CH_2_), 2.04–2.01 (m, 2H, CH_2_CH_2_). ^13^C NMR (100 MHz, CDCl_3_): δ = 152.7, 149.8, 147.7, 144.6, 140.7 (2C), 123.1, 121.1, 115.9, 46.8, 46.4, 26.3, 24.9. HRMS (ESI): *m/z* [M + H]^+^ calcd for C_14_H_15_N_4_O_2_: 271.1190; found: 271.1186.

*N-(5-Nitrosoisoquinolin-6-yl)piperidine-1-carboxamide* (**10c**). Yellow solid; yield: 35.5 mg (25%); mp 198–199 °C (dec., PhMe-EtOAc). ^1^H NMR (400 MHz, CDCl_3_): δ = 13.81 (br s, 1H, NH^…^O), 9.26 (br s, 1H, H-1), 9.21 (d, *J* = 5.3 Hz, 1H, H-3), 8.93 (d, *J* = 9.5 Hz, 1H, H-8), 8.80 (br s, 1H, H-4), 8.19 (d, *J* = 9.5 Hz, 1H, H-7), 3.70 (br s, 4H, N(CH_2_)_2_), 1.75 (br s, 6H, (CH_2_)_3_). ^13^C NMR (100 MHz, CDCl_3_): δ = 153.2, 150.3, 148.0, 145.4, 140.7 (2C), 123.2, 121.1 (2C), 115.7, 29.8, 25.9, 24.3. HRMS (ESI): *m/z* [M + H]^+^ calcd for C_15_H_17_N_4_O_2_: 285.1346; found: 285.1340. HRMS (ESI): *m/z* [M + Na]^+^ calcd for C_15_H_16_N_4_NaO_2_: 307.1163; found: 307.1160.

*N-(5-Nitrosoisoquinolin-6-yl)morpholine-4-carboxamide* (**10d**). Brown solid; yield: 28.6 mg (20%); mp 146–147 °C (dec., PhMe-EtOAc). ^1^H NMR (400 MHz, CDCl_3_): δ = 13.69 (br s, 1H, NH^…^O), 9.32 (s, 1H, H-1), 9.26 (d, 1H, *J* = 6.0 Hz, H-3), 8.97 (d, 1H, *J* = 9.5 Hz, H-8), 8.82 (d, 1H, *J* = 6.0 Hz, H-4), 8.26 (d, 1H, *J* = 9.5 Hz, H-7), 3.83-3.87 (m, 4H, O(CH_2_)_2_), 3.74–3.79 (m, 4H, N(CH_2_)_2_). ^13^C NMR (100 MHz, CDCl_3_): δ = 153.7, 150.3, 148.0, 145.3, 141.2 (2C), 129.7, 123.4, 120.8, 115.9, 66.5, 44.6. HRMS (ESI): *m/z* [M + H]^+^ calcd for C_14_H_15_N_4_O_3_: 287.1139; found: 287.1134.

*1,1-Dialkyl-3-(5-nitroisoquinolin-8-yl)ureas (**11a***–***d****); (General Procedure):*

To a solution of 2 mmol of the corresponding urea (Scheme 5, Method B) in 8 mL of DMSO, containing 0.5% water, 112 mg of KOH (2 mmol), 87 mg (0.5 mmol) of 5-nitroisoquinoline and 658 mg (2 mmol) K_3_Fe(CN)_6_ were added sequentially at room temperature. The mixture was intensively stirred at room temperature for 4 h, then the reaction mass was poured into 50 g of ice and, upon reaching room temperature, it was acidified with dilute HCl to pH~7. The aqueous solution was extracted with ethyl acetate (4 × 10 mL), the extract was dried with Na_2_SO_4_. After evaporation of the solvent, the dry residue was purified by dry flash chromatography on silica gel,^32^ collecting in all cases the second yellow fraction, from which compounds **11a**–**d** were obtained. Eluents: PhMe- EtOAc (10:1) for **11a**; PhMe- EtOAc (3:2) for **11b**; PhMe- EtOAc (1:1) for **11c**; ethyl EtOAc for **11d**.

*1,1-Dimethyl-3-(5-nitroisoquinolin-8-yl)urea* (**11a**). Yellow solid; yield: 46.8 mg (36%); mp 218–219 °C (dec., PhMe-EtOAc). ^1^H NMR (400 MHz, DMSO-d_6_): δ = 9.58 (s, 1H, H-1), 9.25 (br s, 1H, NH), 8.73 (d, *J* = 6.1 Hz, 1H, H-3), 8.66 (d, *J* = 8.8 Hz, 1H, H-6), 8.46 (d, *J* = 6.1 Hz, 1H, H-4), 7.90 (d, *J* = 8.8 Hz, 1H, H-7), 3.07 (s, 6H, N(CH_3_)_2_). ^13^C NMR (100 MHz, DMSO-*d*_6_): δ = 155.4, 149.4, 146.2, 144.8, 138.0, 130.4, 128.8, 121.0, 117.9, 115.1, 36.6. HRMS (ESI): *m/z* [M + H]^+^ calcd for C_12_H_13_N_4_O_3_: 261.0982; found: 261.0979. HRMS (ESI): *m/z* [M + Na]^+^ calcd for C_12_H_12_N_4_NaO_3_: 283.0785; found: 283.0792.

*N-(5-Nitroisoquinolin-8-yl)pyrrolidine-1-carboxamide* (**11b**). Yellow solid; yield: 32.9 mg (23%); mp 197–198 °C (dec., PhMe-EtOAc). ^1^H NMR (400 MHz, DMSO-d_6_): δ = 9.61 (br s, 1H, H-1), 9.10 (br s, 1H, NH), 8.74 (dd, *J* = 6.2, 0.8 Hz, 1H, H-3), 8.66 (dd, *J* = 8.8, 0.7 Hz, 1H, H-6), 8.47 (d, *J* = 6.2 Hz, 1H, H-4), 8.03 (br d, *J* = 8.8 Hz, 1H, H-7), 3.54 (br s, 4H, N(CH_2_)_2_), 1.92 (br s, 4H, (CH_2_)_2_). ^13^C NMR (100 MHz, DMSO-*d*_6_): δ = 153.3, 149.4, 146.3, 144.4, 137.9, 130.5, 128.9, 120.8, 117.7, 115.1, 46.1, 25.1. HRMS (ESI): *m/z* [M + H]^+^ calcd for C_14_H_14_N_4_O_3_: 287.1139; found: 287.1126.

*N-(5-Nitroisoquinolin-8-yl)piperidine-1-carboxamide* (**11c**). Yellow solid; yield: 52.5 mg (35%); mp 205–206 °C (dec., PhMe-EtOAc). ^1^H NMR (400 MHz, DMSO-d_6_): δ = 9.54 (br s, 1H, H-1), 9.44 (br s, 1H, NH), 8.73 (d, *J* = 6.1 Hz, 1H, H-3), 8.63 (d, *J* = 8.8 Hz, 1H, H-6), 8.47 (d, *J* = 6.1 Hz, 1H, H-4), 7.83 (d, *J* = 8.8 Hz, 1H, H-7), 3.55 (t, *J* = 5.2 Hz, 4H, N(CH_2_)_2_), 1.63-1.56 (m, 6H, (CH_2_)_3_). ^13^C NMR (100 MHz, DMSO-*d*_6_): δ = 154.6, 149.4, 146.3, 145.2, 137.9, 130.5, 128.9, 121.0, 117.8, 115.1, 45.2, 25.6, 24.0. HRMS (ESI): *m/z* [M + Na]^+^ calcd for C_15_H_16_N_4_NaO_3_: 323.1115; found: 323.1105.

*N-(5-Nitroisoquinolin-8-yl)morpholine-4-carboxamide* (**11d**). Yellow solid; yield: 58.9 mg (39%); mp 230–231 °C (dec., PhMe-EtOAc). ^1^H NMR (400 MHz, DMSO-d_6_): δ = 9.59 (s, 1H, H-1), 9.47 (br s, 1H, NH), 8.74 (d, 1H, *J* = 6.1 Hz, H-3), 8.66 (d, 1H, *J* = 8.8 Hz, H-6), 8.46 (d, 1H, *J* = 6.1 Hz, H-4), 7.90 (d, 1H, *J* = 8.8 Hz, H-7), 3.68 (t, 4H, *J* = 4.5 Hz, O(CH_2_)_2_), 3.56 (t, 4H, *J* = 4.5 Hz, N(CH_2_)_2_). ^13^C NMR (100 MHz, DMSO-*d*_6_): δ = 154.9, 149.4, 146.3, 144.6, 138.2, 130.4, 128.8, 121.1, 118.1, 115.1, 66.0, 44.6. HRMS (ESI): *m/z* [M + H]^+^ calcd for C_14_H_15_N_4_O_4_: 303.1088; found: 303.1089.

## 4. Conclusions

Thus, different regioselectivity was found in the reactions of S_N_^H^ substitution of 5-nitroisoquinoline with N-anions of aromatic amides and ureas, depending on the absence or presence of small amounts of water in the reaction mass. So, interaction with amides in anhydrous DMSO usually results in the formation of a mixture of hitherto unknown 8-aroylamino-5-nitroisoquinolines and 6-aroylamino-5-nitrosoisoquinolines in a small or moderate yield. In the presence of water, only nitro derivatives were formed. Anions of 1,1-dialkylureas in anhydrous DMSO give rise to 6-dialkylcarbamoylamino-5-nitrosoisoquinolines, whereas in the presence of water, 8-dialkylcarbamoylamino-5-nitroisoquinolines form. Urea itself and its monosubstituted derivatives under anhydrous conditions form exclusively 5-nitrosoisoquinoline-6-amine.

## Figures and Tables

**Table 1:** Optimization of the Reaction Conditions of S_N_^H^ Amidation of 5-Nitroisoquinoline (**1**) with the *p*-Methylbenzamide N-anion in anhydrous DMSO (Method A).

Entry	Reaction Time, h	Excess of NaH, Equiv	Excess of Amide, Equiv	Yield, % *^a^*	
				2a	3a
1 *^b^*	1.5	2	2	19	26
2 *^b^*	1.5	4	4	30	traces
3 *^b^*	1.5	6	6	24	7.5
4 *^b^*	1.5	1	3	45	traces
5 *^b^*	1.5	4	2	28	15
6 *^b,c^*	1.5	2	2	28	traces
7 *^d^*	0.5	2	2	40	traces
8 *^b,e^*	1.5	2	2	15	23

*^a^* Isolated yields after column chromatography. *^b^* The experiment was performed at room temperature. *^c^* The experiment was performed in presence of K_3_Fe(CN)_6_. *^d^* The experiment was performed at 60 °C. *^e^* The reaction was performed under argon.

## Data Availability

Supporting Information data include NMR spectral charts.

## Supplementary Materials

Crystallographic data for the structures in this paper have been deposited in the Cambridge Crystallographic Data Center as a supplementary publication (**2a**, CCDC 2159575; **3a,** CCDC 2159573; **10a,** CCDC 2159579, www.ccdc.cam.ac.uk/getstructures). Copies of the data can be obtained, free of charge, on application to CCDC, 12 Union Road, Cambridge CB12 1EZ, U.K [Fax: +44 1223 336033 or e-mail: deposit@ccdc.cam.ac.uk. The Supplemental Material file of CCDC includes the CIF file of **2a, 3a**, **10a**. The following supporting information can be downloaded at: https://www.mdpi.com/article/10.3390/molecules27227862/s1, NMR spectroscopy data and X-ray analysis.

## Sample Availability

Samples of the all compounds are available from the authors.
