# Supplementary material for: SNH Amidation of 5-Nitroisoquinoline: Access to Nitro- and Nitroso Derivatives of Amides and Ureas on the Basis of Isoquinoline
Source: Molecules. 2022 Nov 14;27(22):7862. doi: 10.3390/molecules27227862 (PMC9694180; doi:10.3390/molecules27227862)
Supplement: Supplementary file 1 [file molecules-27-07862-s001.zip › molecules-2023797-supplementary.pdf]

Supplementory material for the article: S_N^H Amidation of 5-nitroisoquinoline: access to nitro- and nitroso derivatives of amides and ureas on the basis of isoquinoline

Authors: Elena K. Avakyan, Anastasia A. Borovleva, Diana Yu. Pobedinskaya,

Oleg P. Demidov, Artem P. Ermolenko, Alexander N. Larin, Ivan V. Borovlev*

4-Methyl-*N*-(5-nitroisoquinolin-8-yl)benzamide (2a)

NMR ^1H

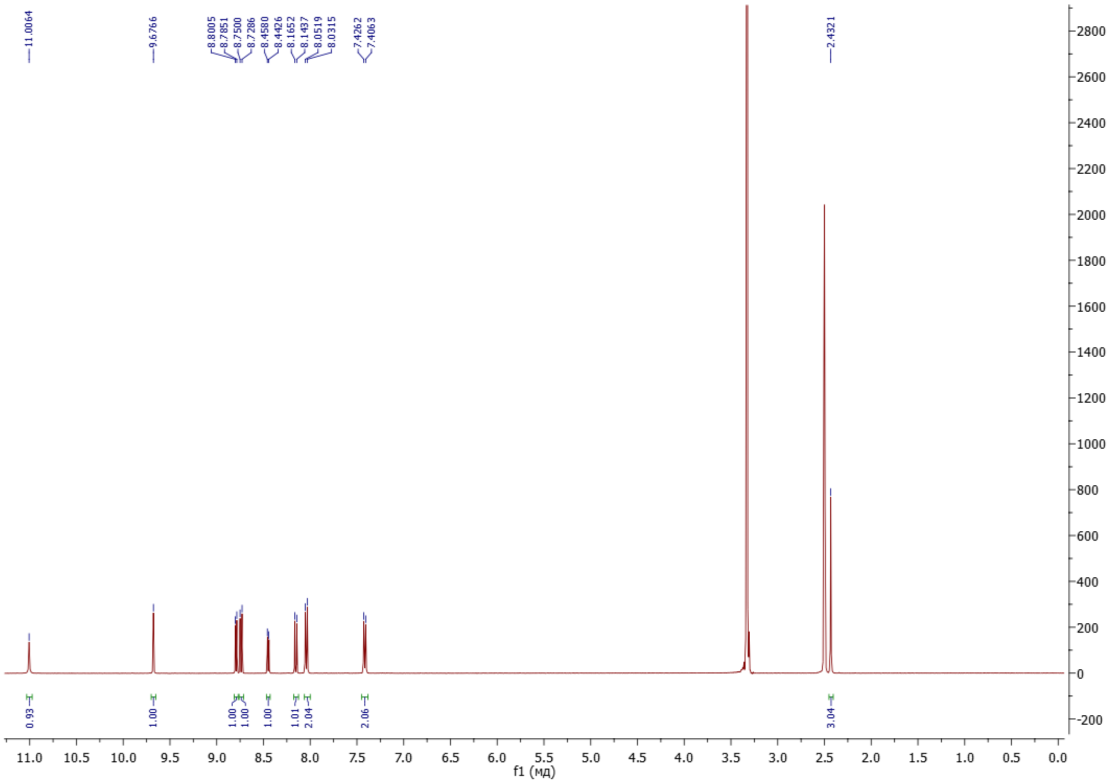

NMR ^{13}C

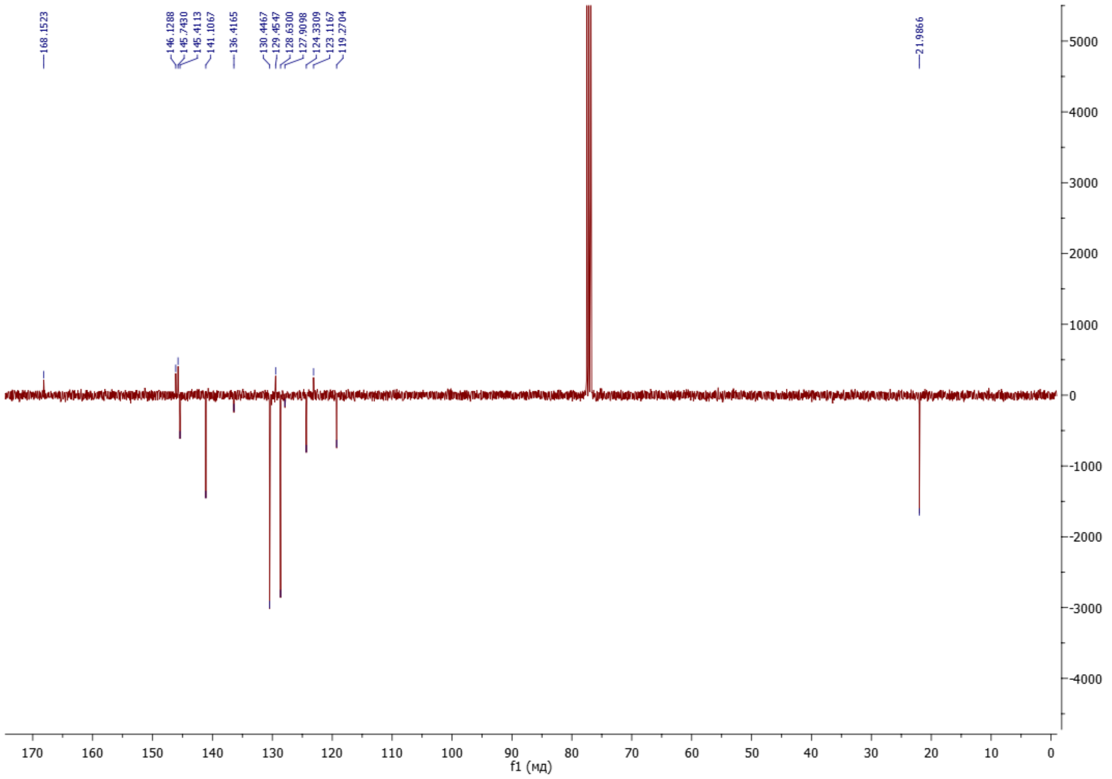

4-Methyl-*N*-(5-nitrosoisoquinolin-6-yl)benzamide (3a)

NMR ^{1}H

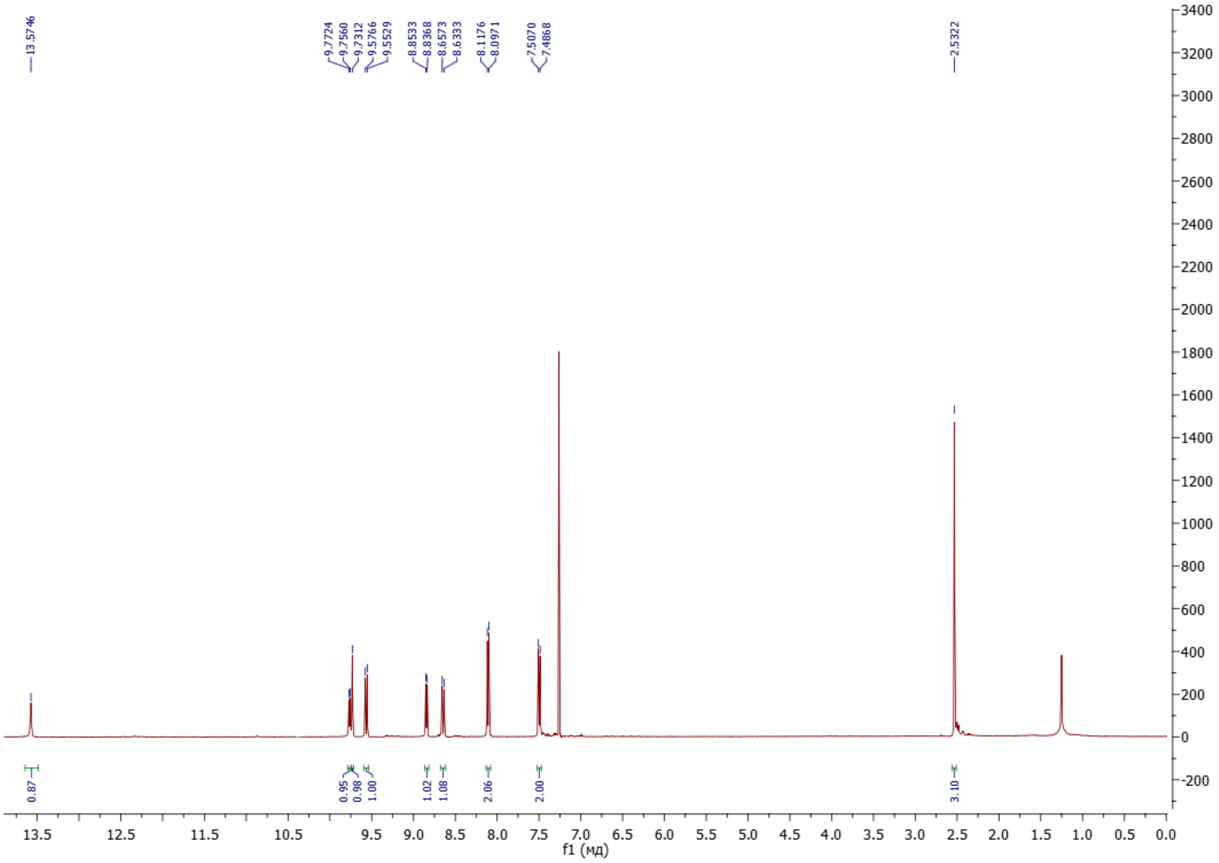

NMR ^{13}C

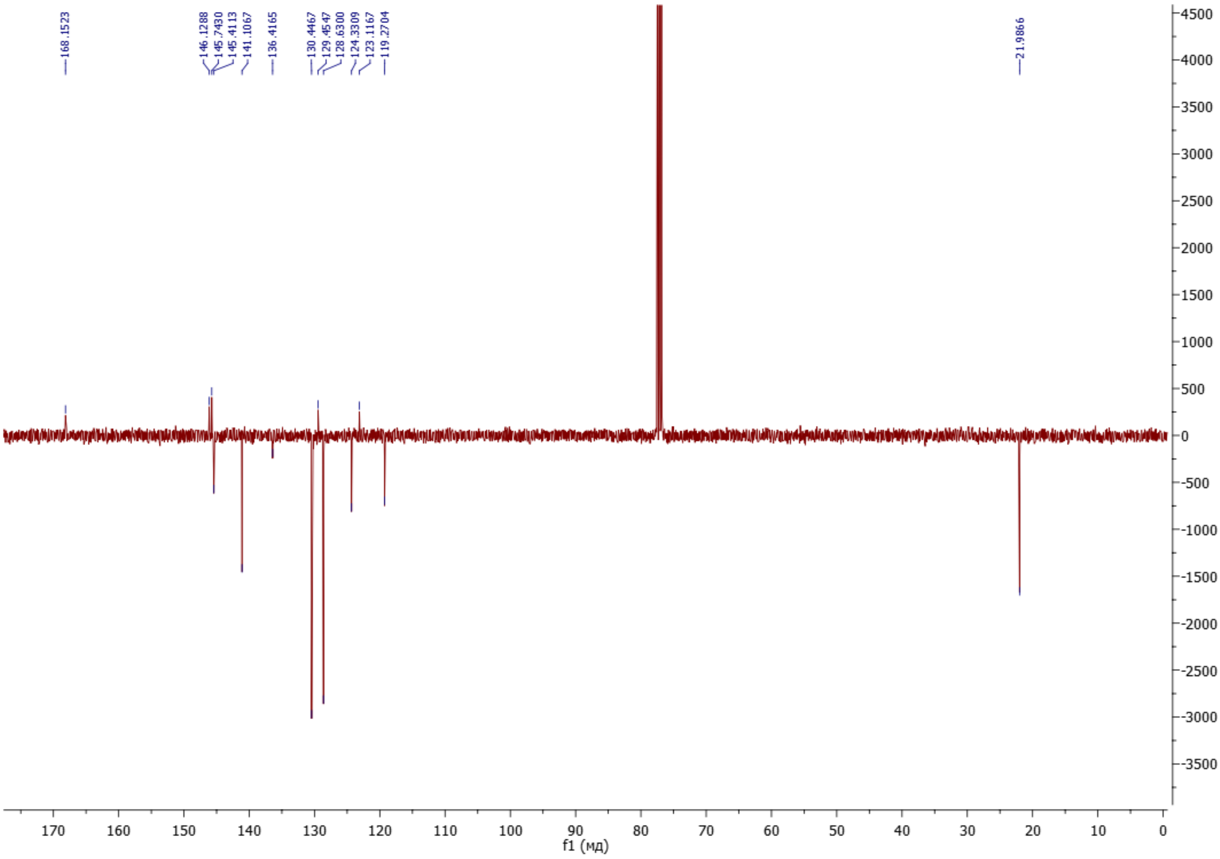

N-(5-Nitroisoquinolin-8-yl)benzamide (2b)

NMR *^{1}H*

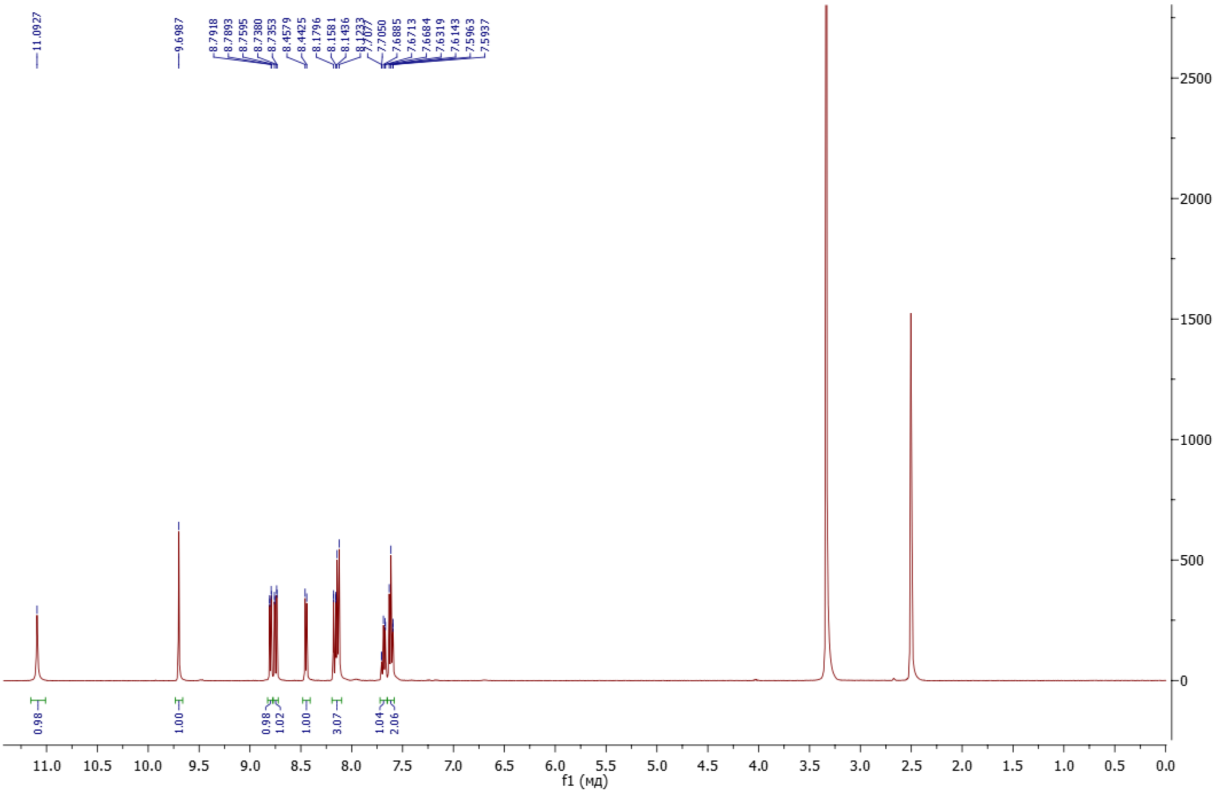

NMR *^{13}C*

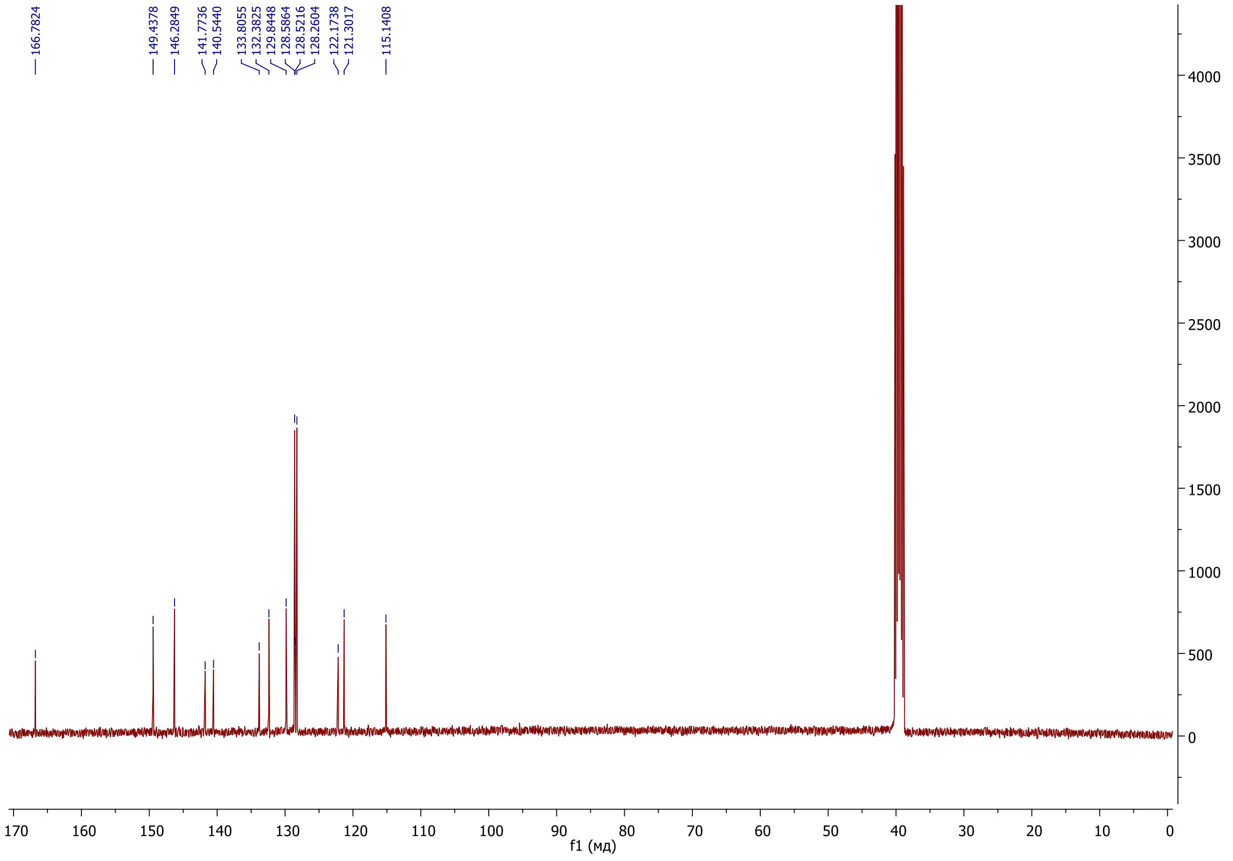

N-(5-Nitrosoisoquinolin-6-yl)benzamide (3b)

NMR ^{1}H

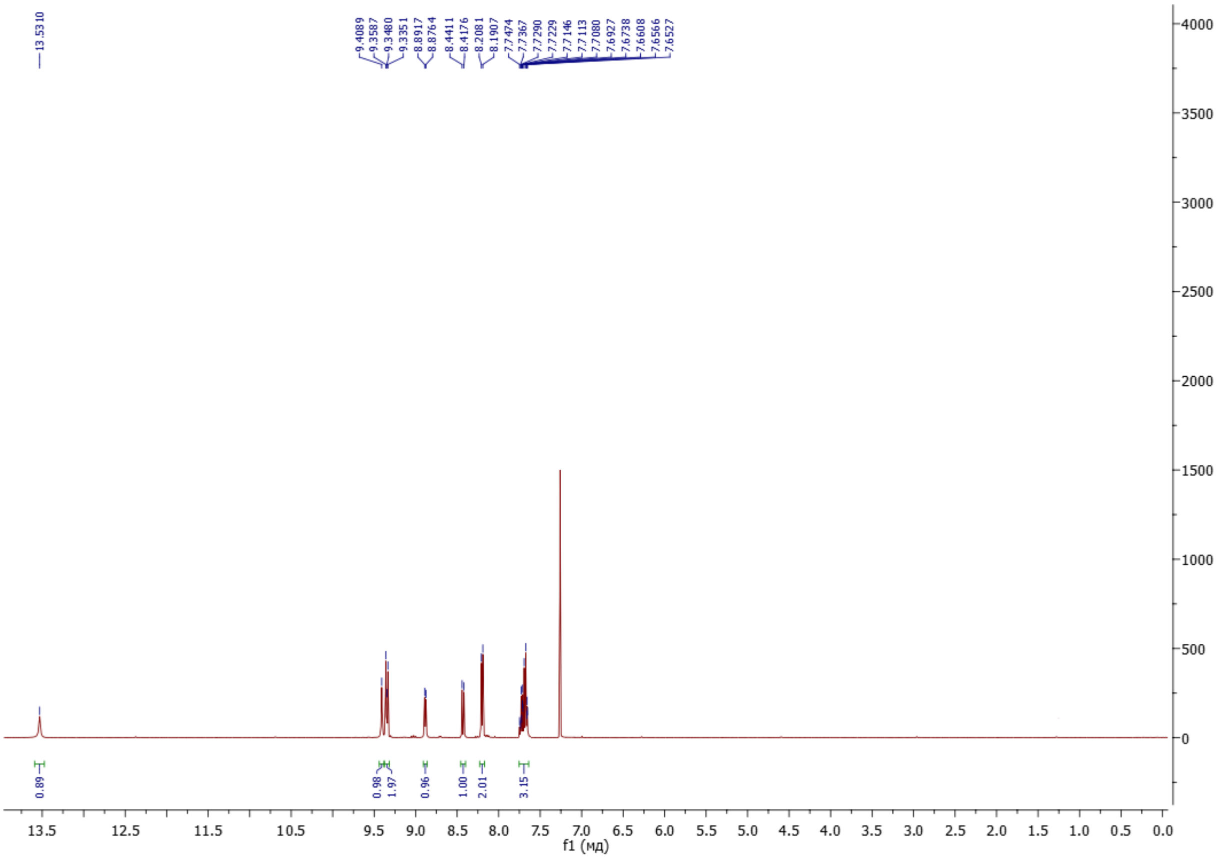

NMR ^{13}C

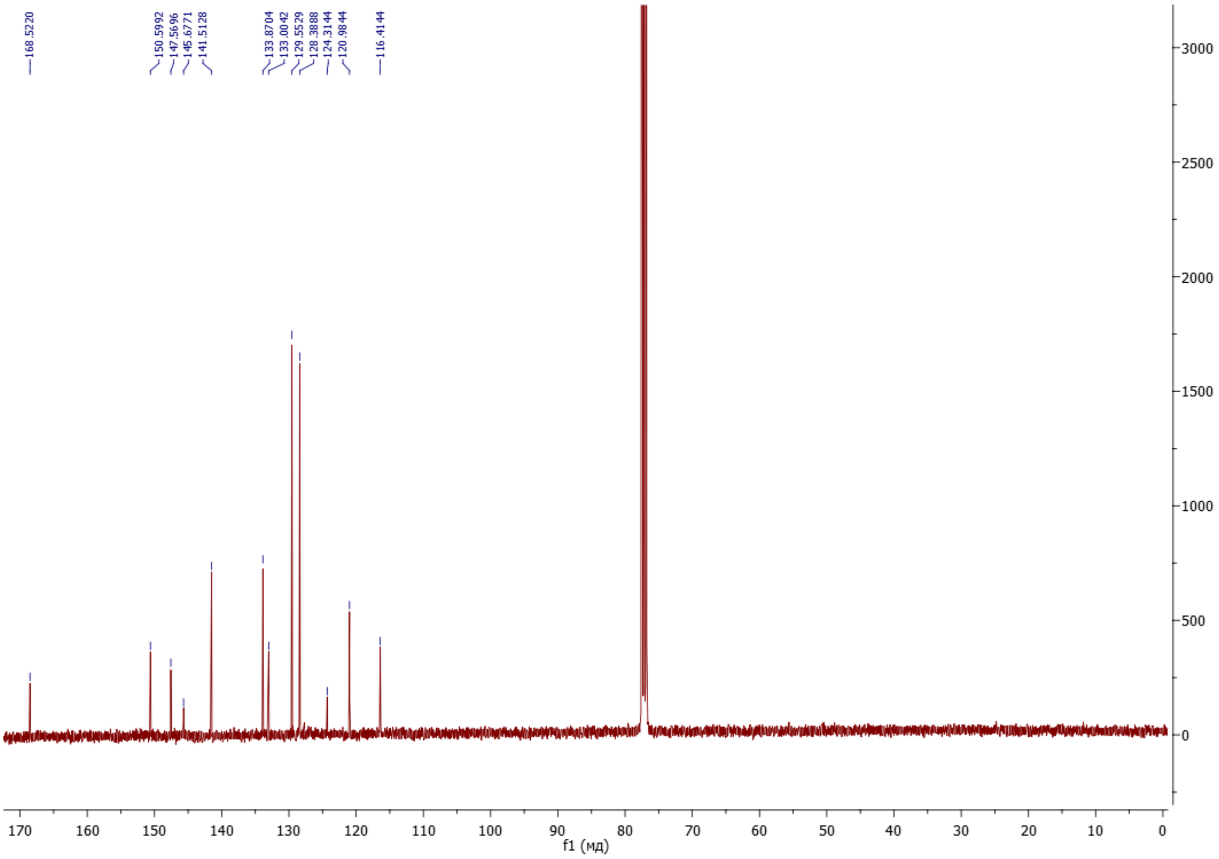

4-Methoxy-*N*-(5-nitroisoquinolin-8-yl)benzamide (2c)

NMR ^1H

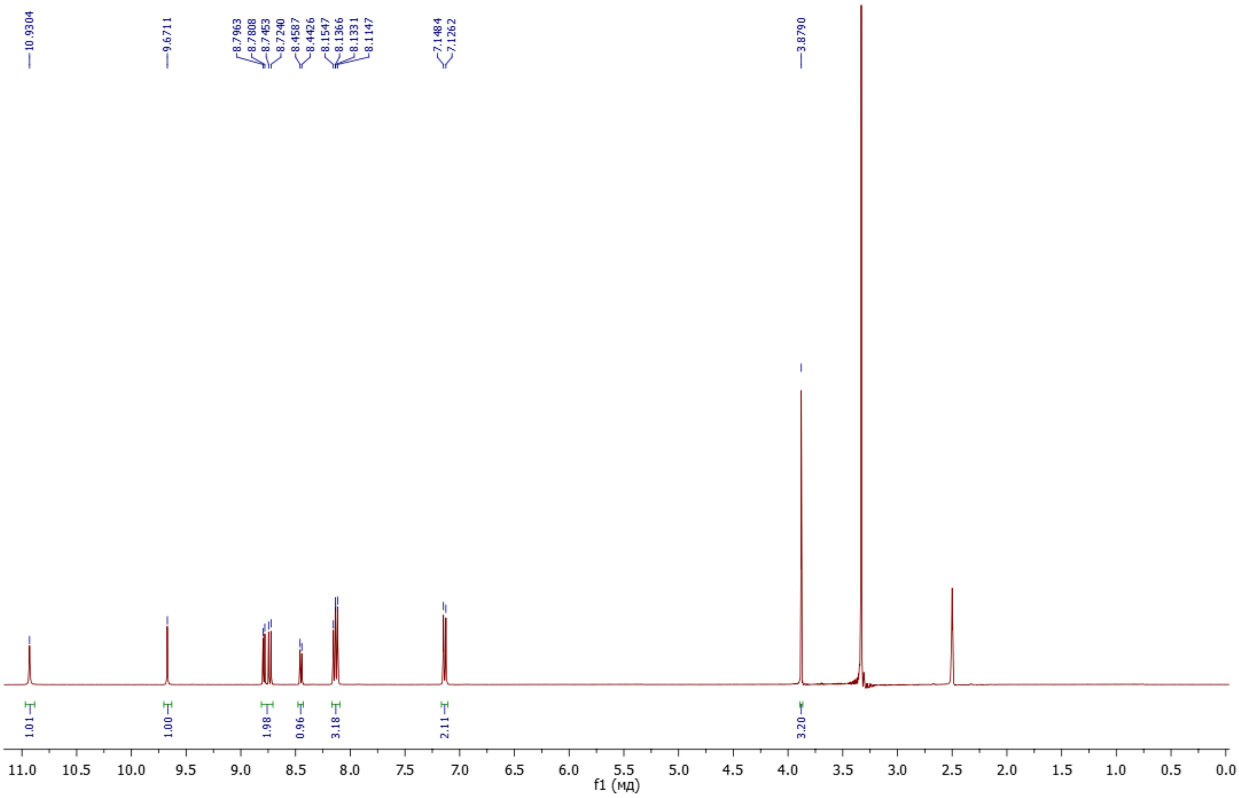

NMR ^{13}C

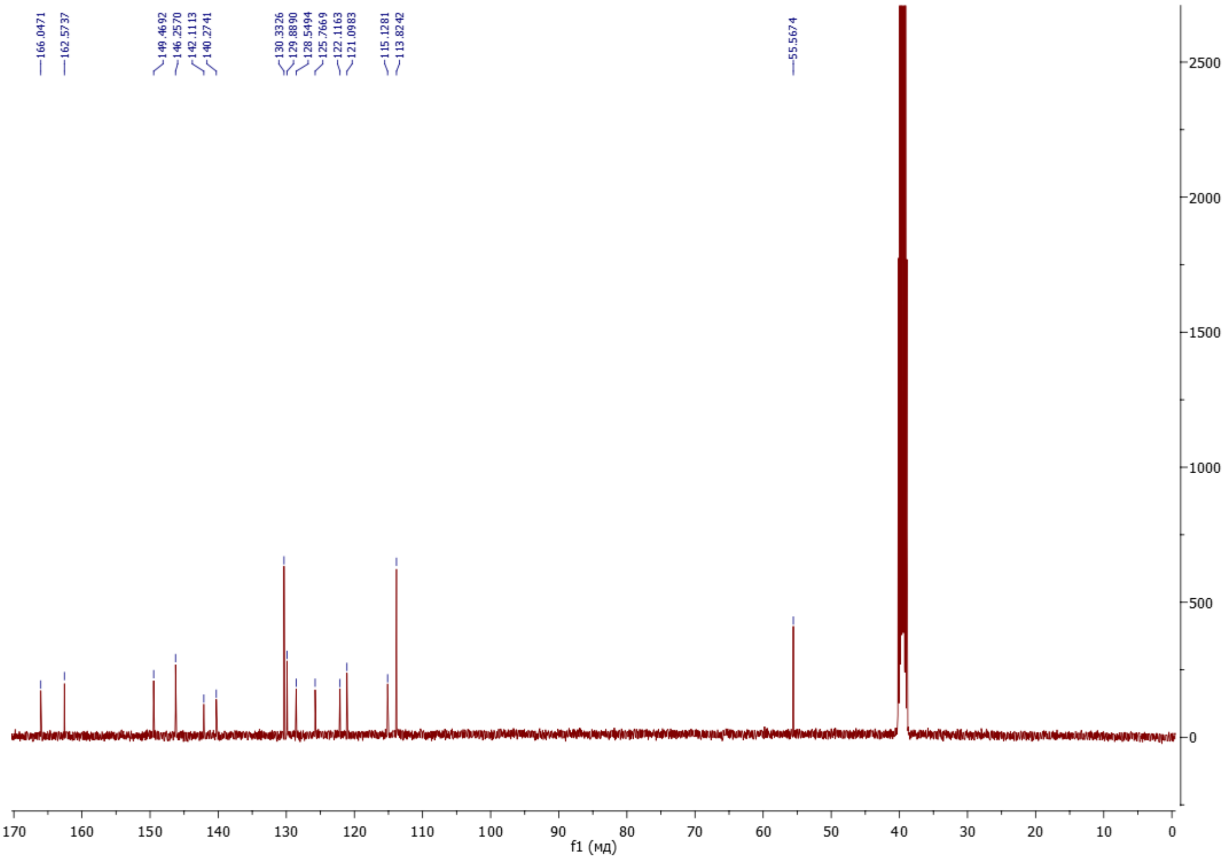

4-Methoxy-*N*-(5-nitrosoisoquinolin-6-yl)benzamide (3c)

NMR ^{1}H

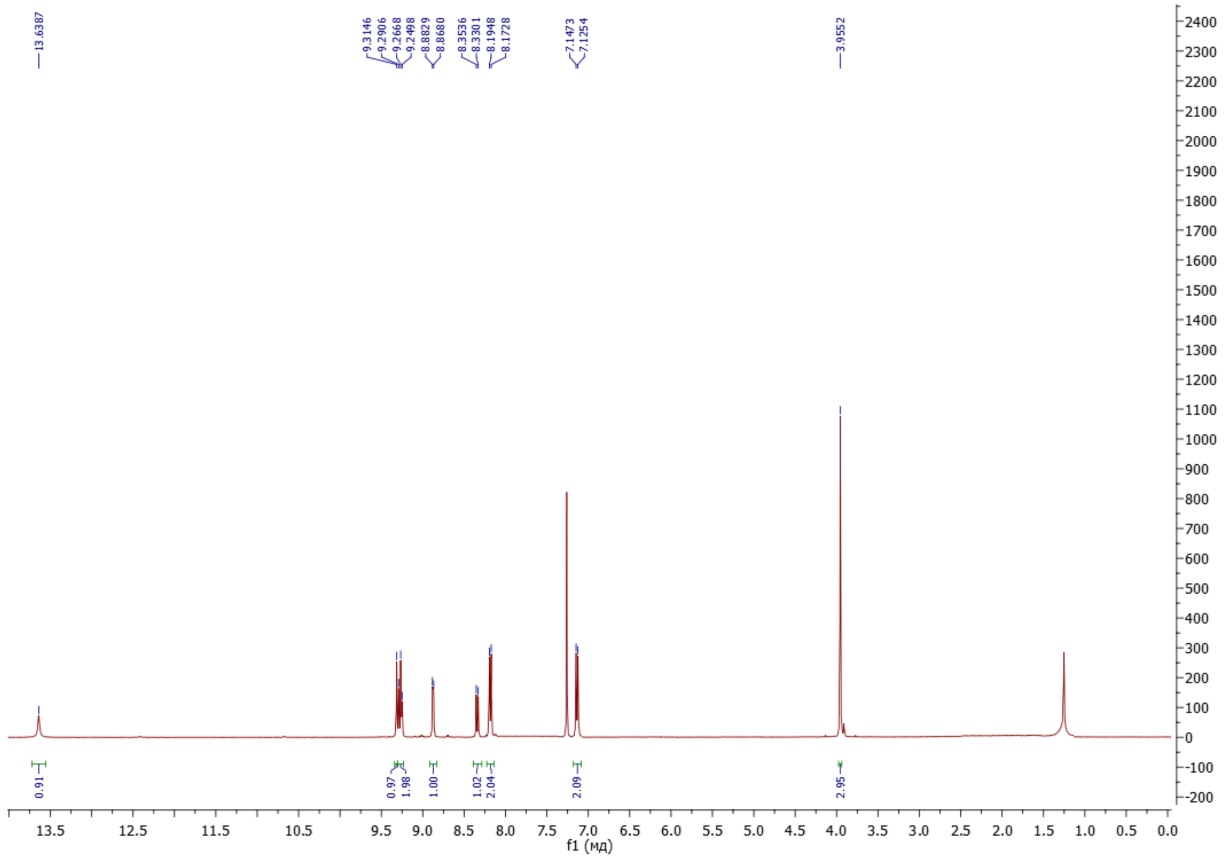

NMR ^{13}C

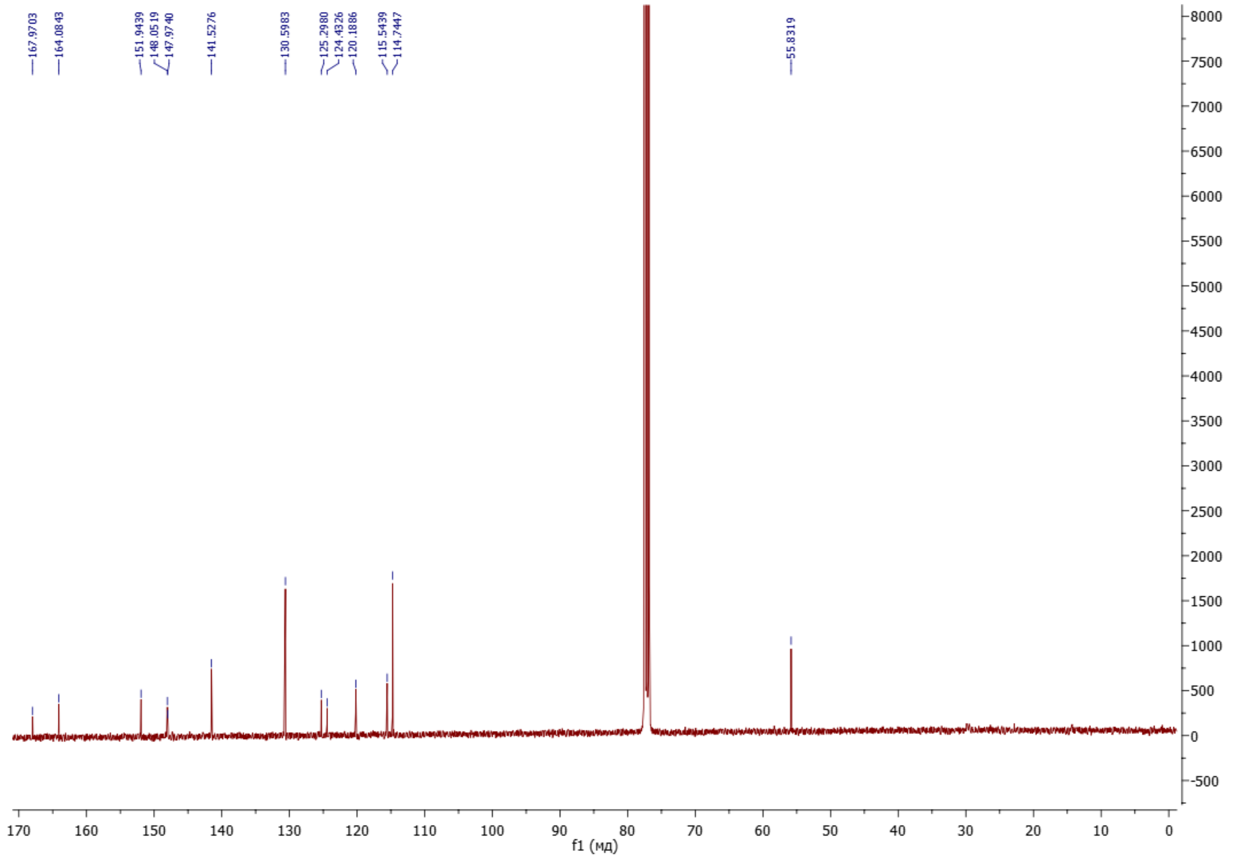

4-Nitro-*N*-(5-nitroisoquinolin-8-yl)benzamide (2d)

NMR ^1H

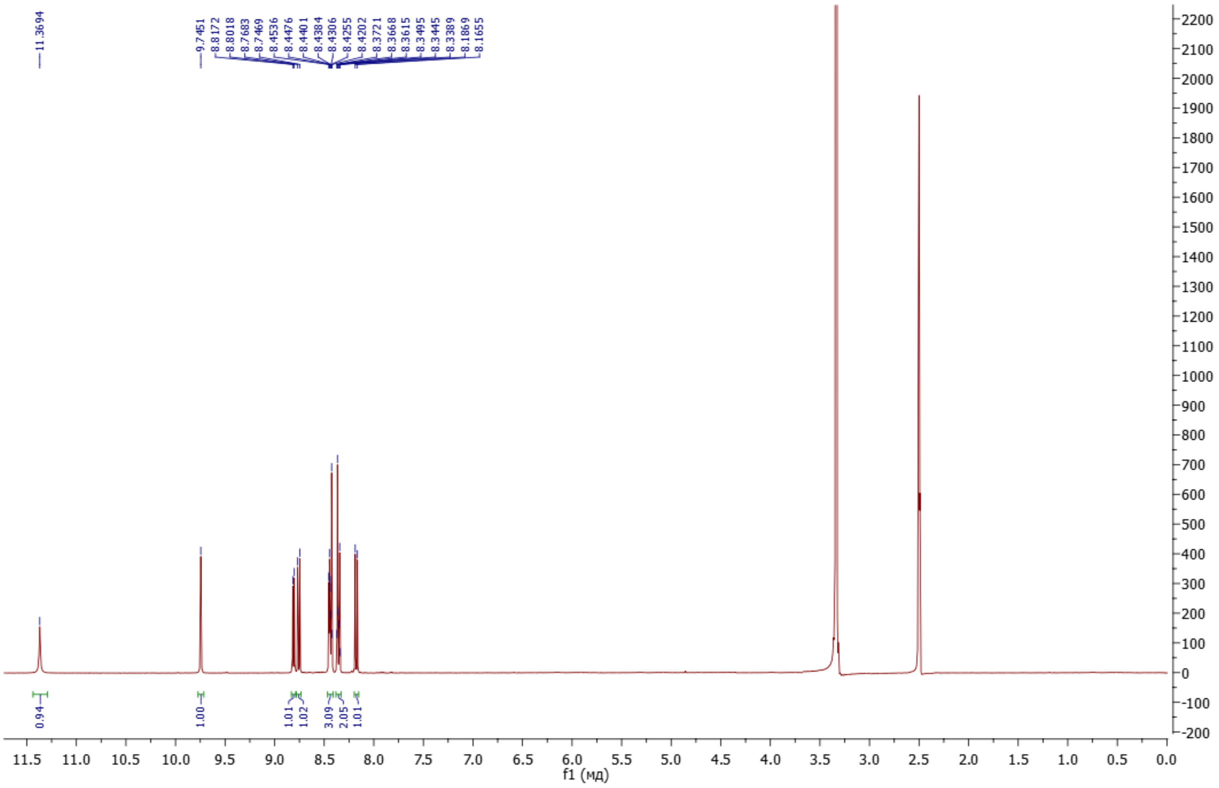

NMR ^{13}C

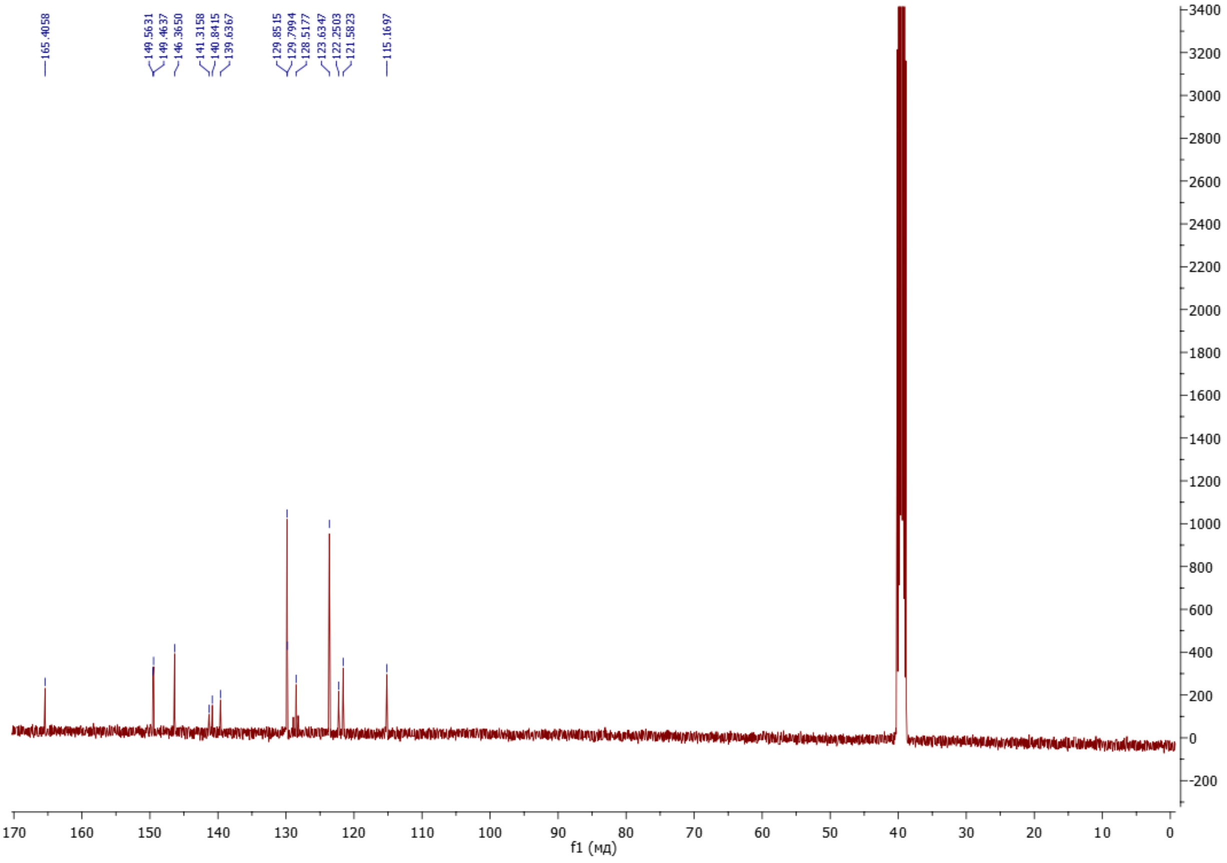

3-Nitro-*N*-(5-nitroisoquinolin-8-yl)benzamide (2e)

NMR ^1H

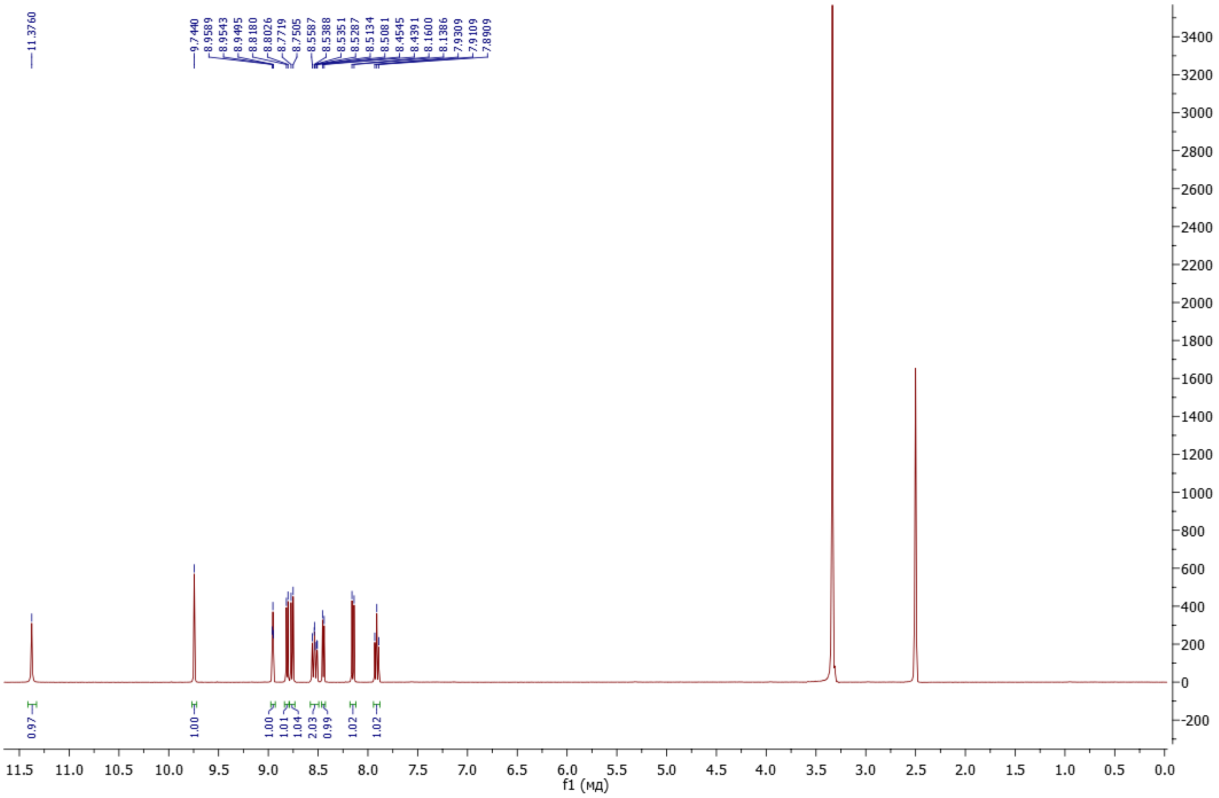

NMR ^{13}C

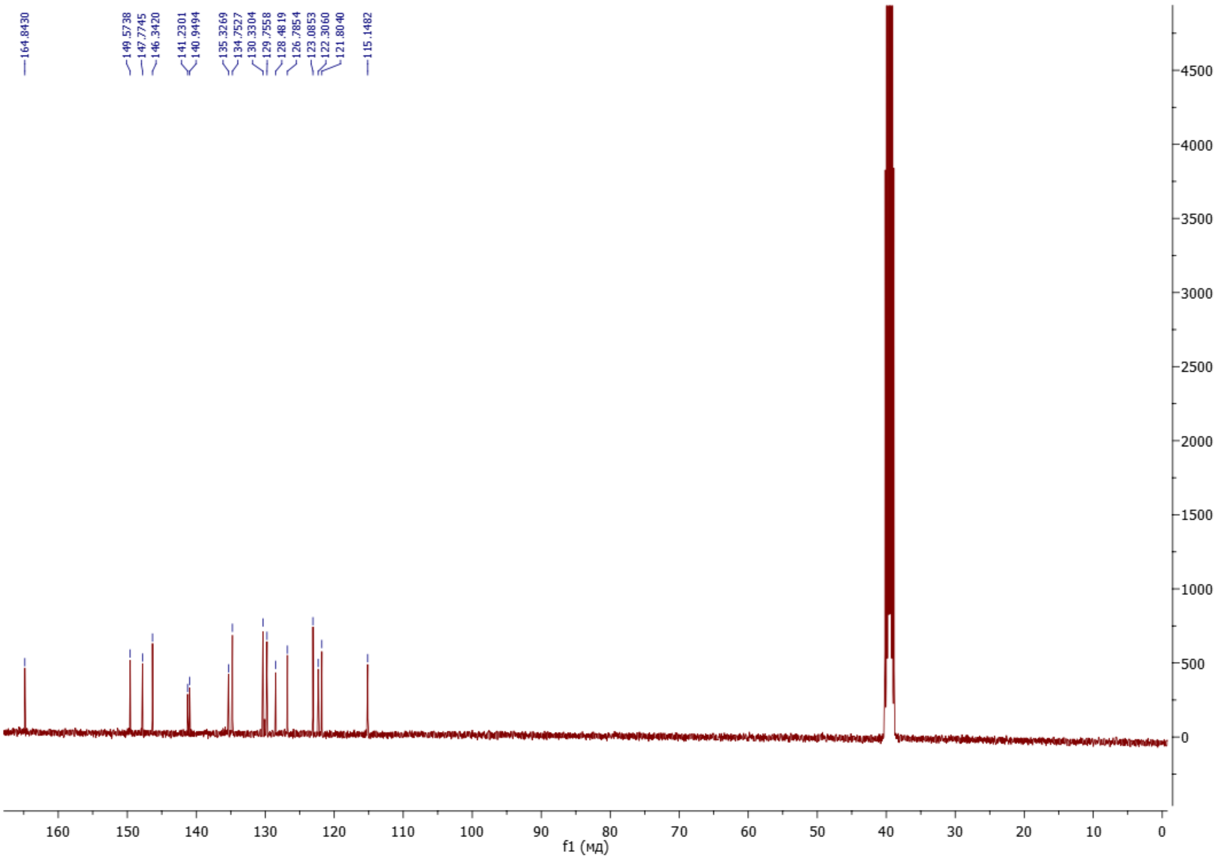

2-Nitro-*N*-(5-nitroisoquinolin-8-yl)benzamide (2f)

NMR ¹H

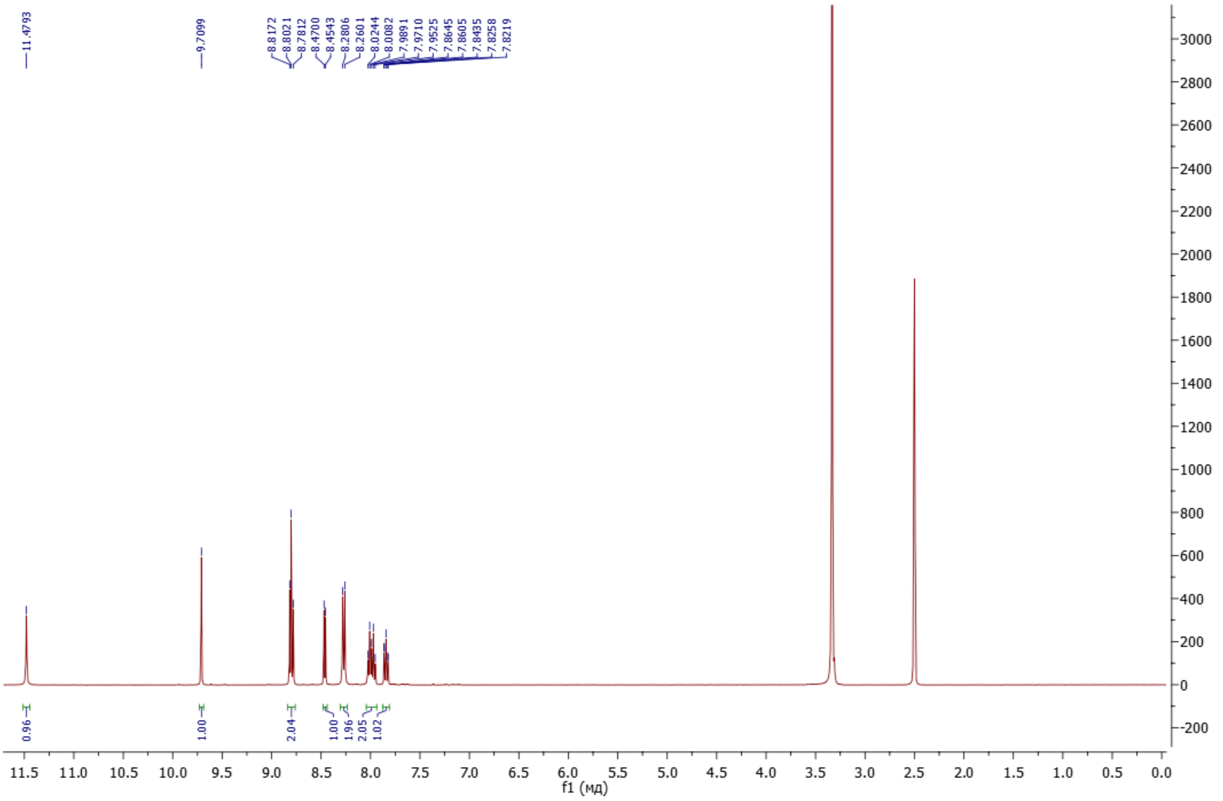

NMR ¹³C

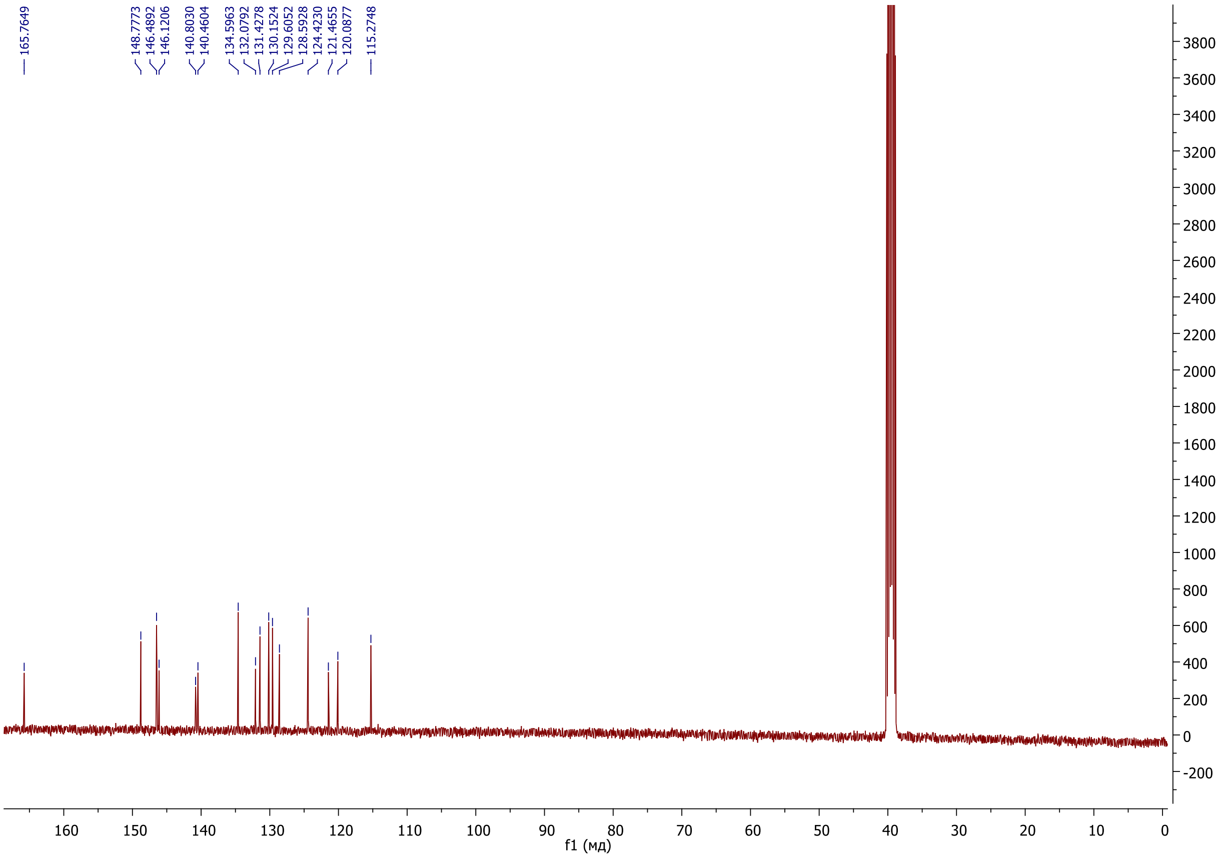

5-Nitrosoisoquinolin-6-amine (7).

NMR ^{1}***H***

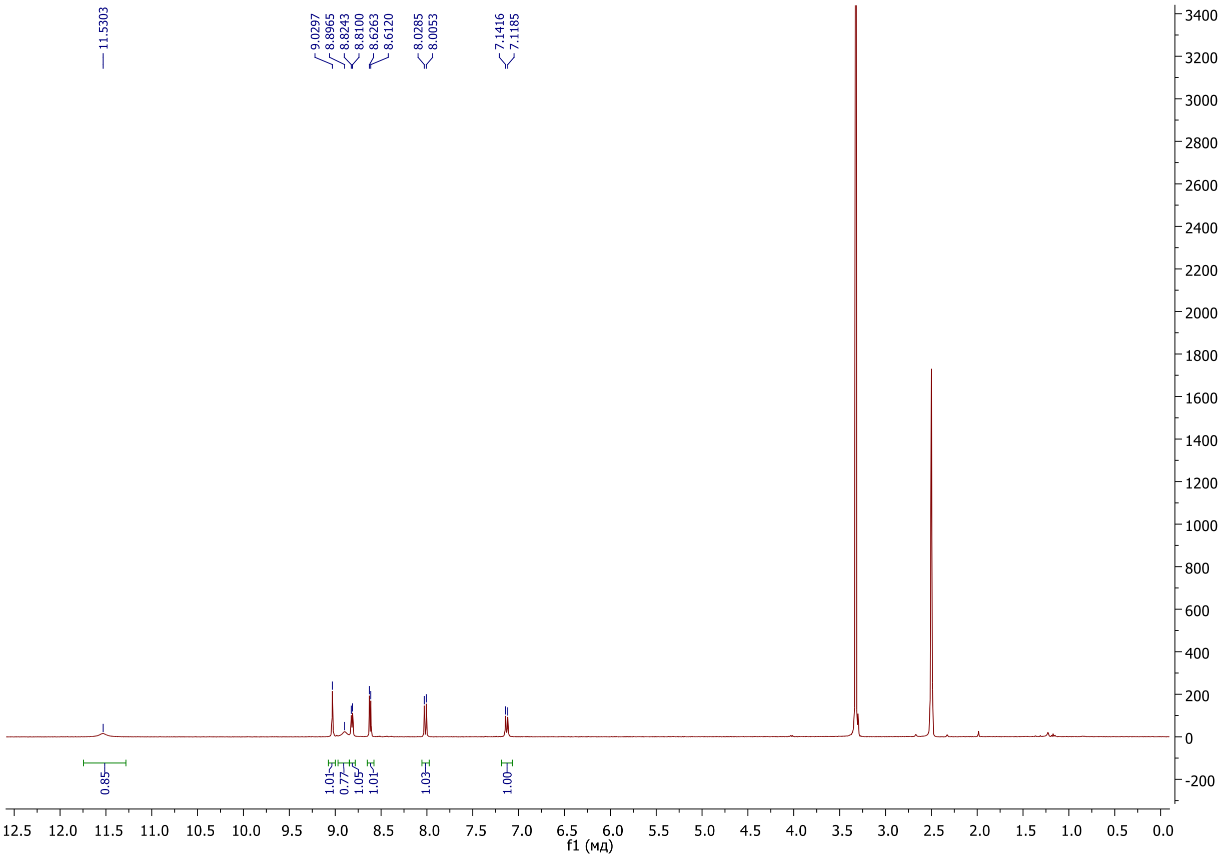

NMR ^{13}***C***

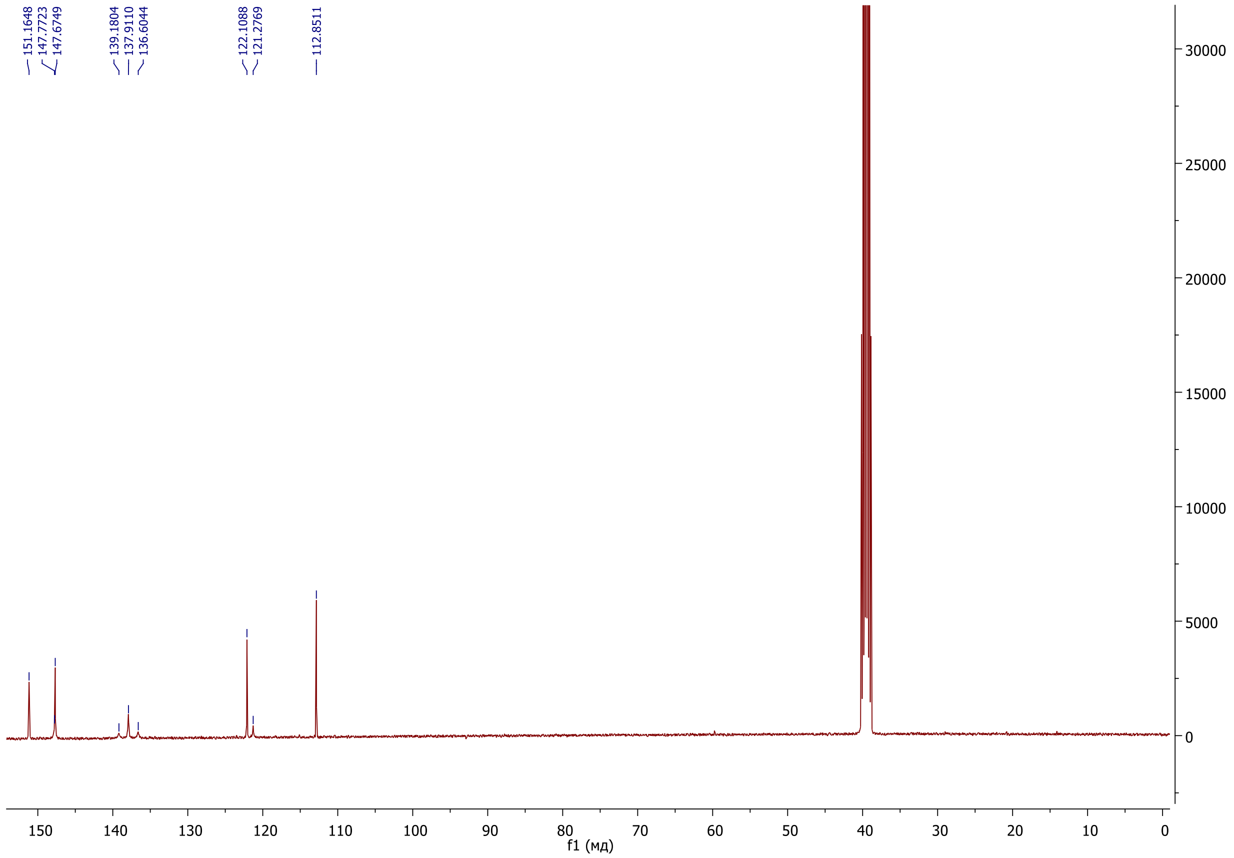

1,1-Dimethyl-3-(5-nitrosoisoquinolin-6-yl)urea (10a)

NMR ^{1}H at 25 ^{o}C

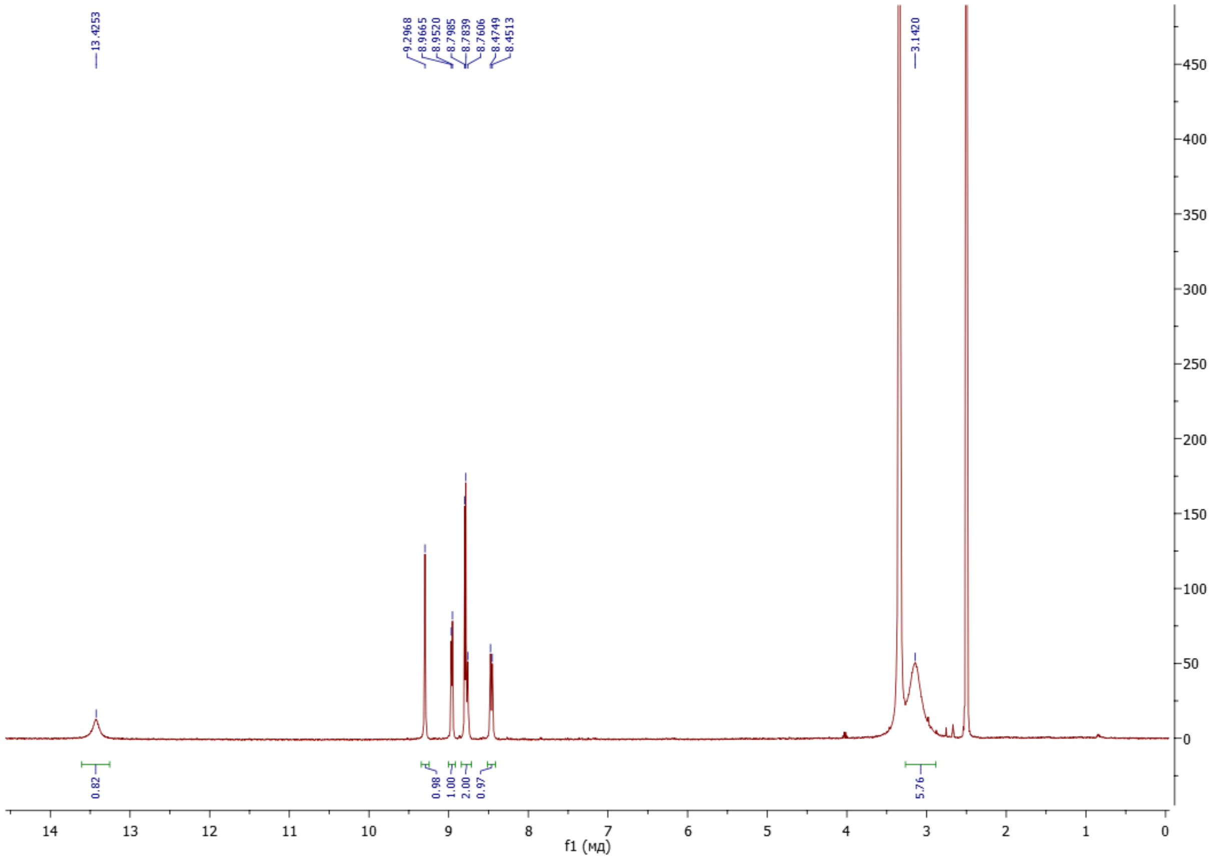

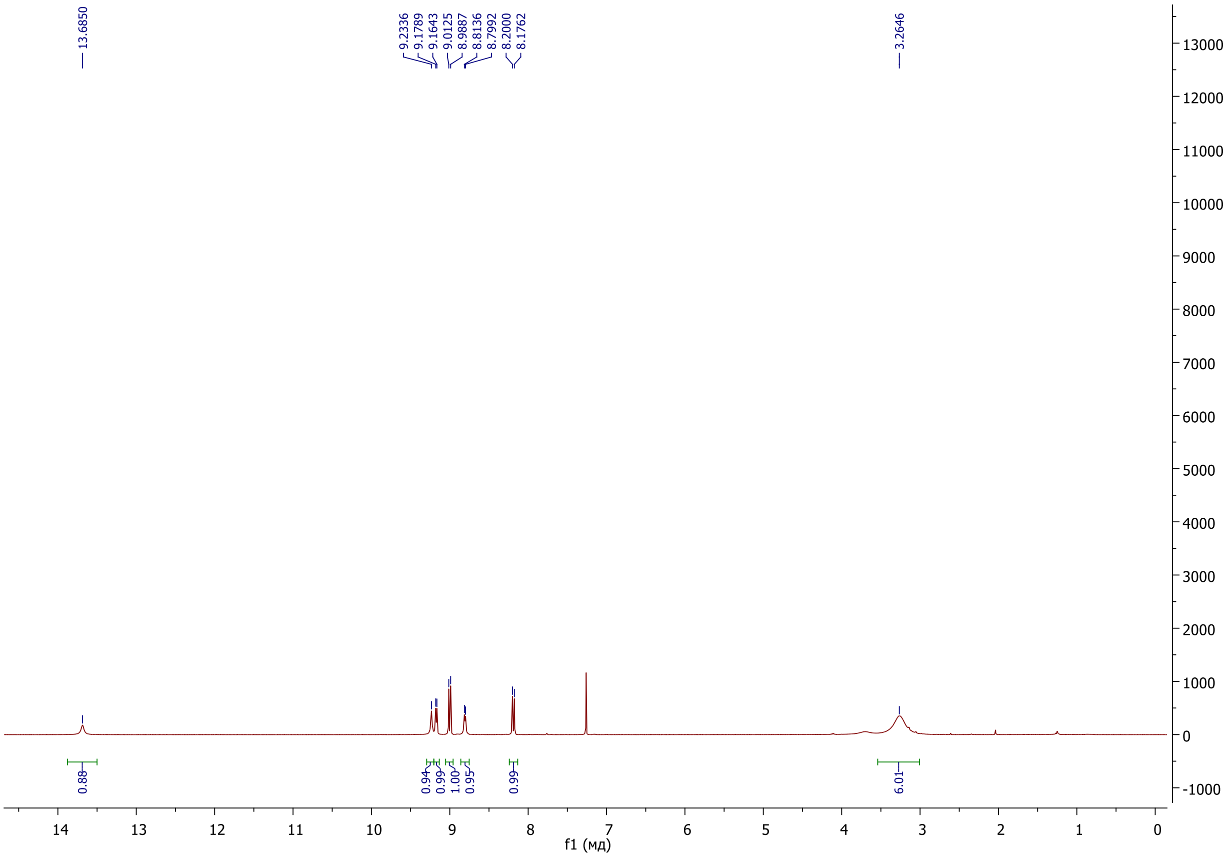

NMR ^{1}H at 13,7 ^{o}C

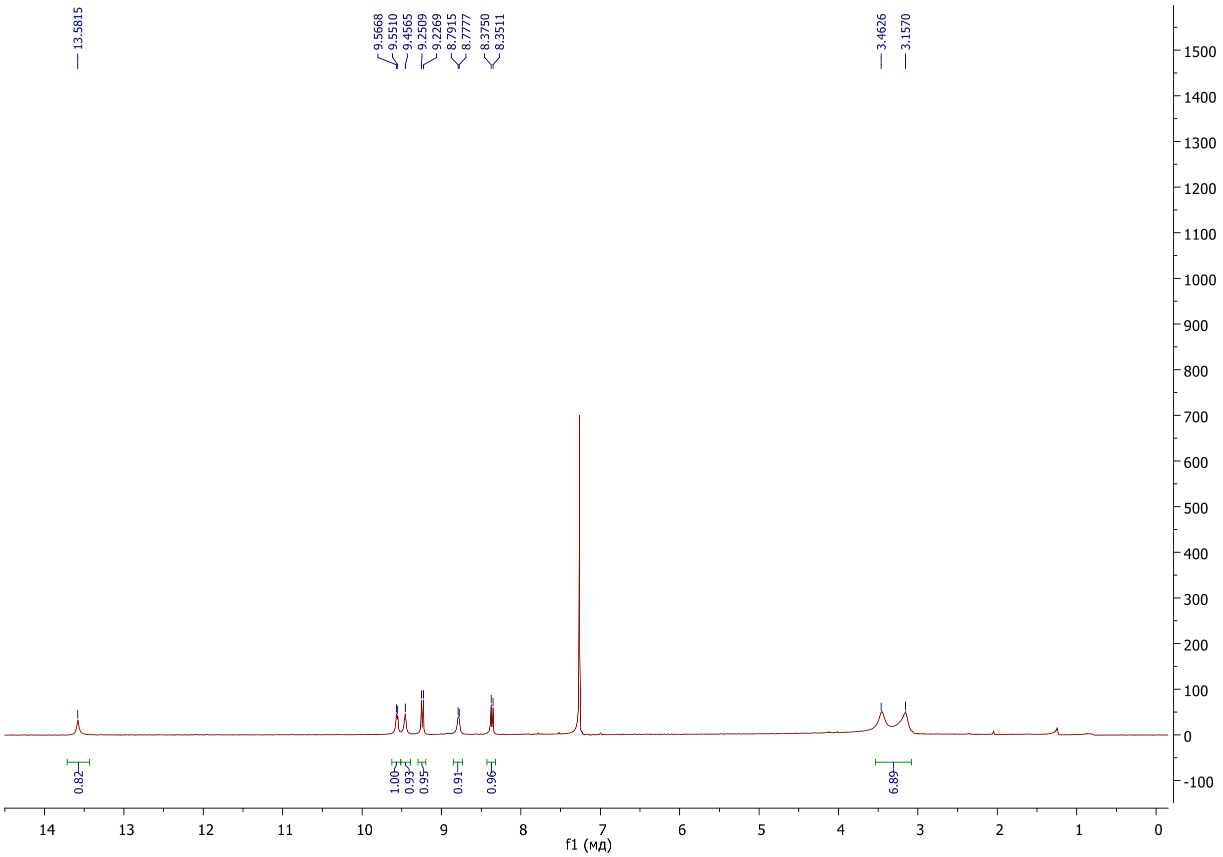

NMR ^{13}C

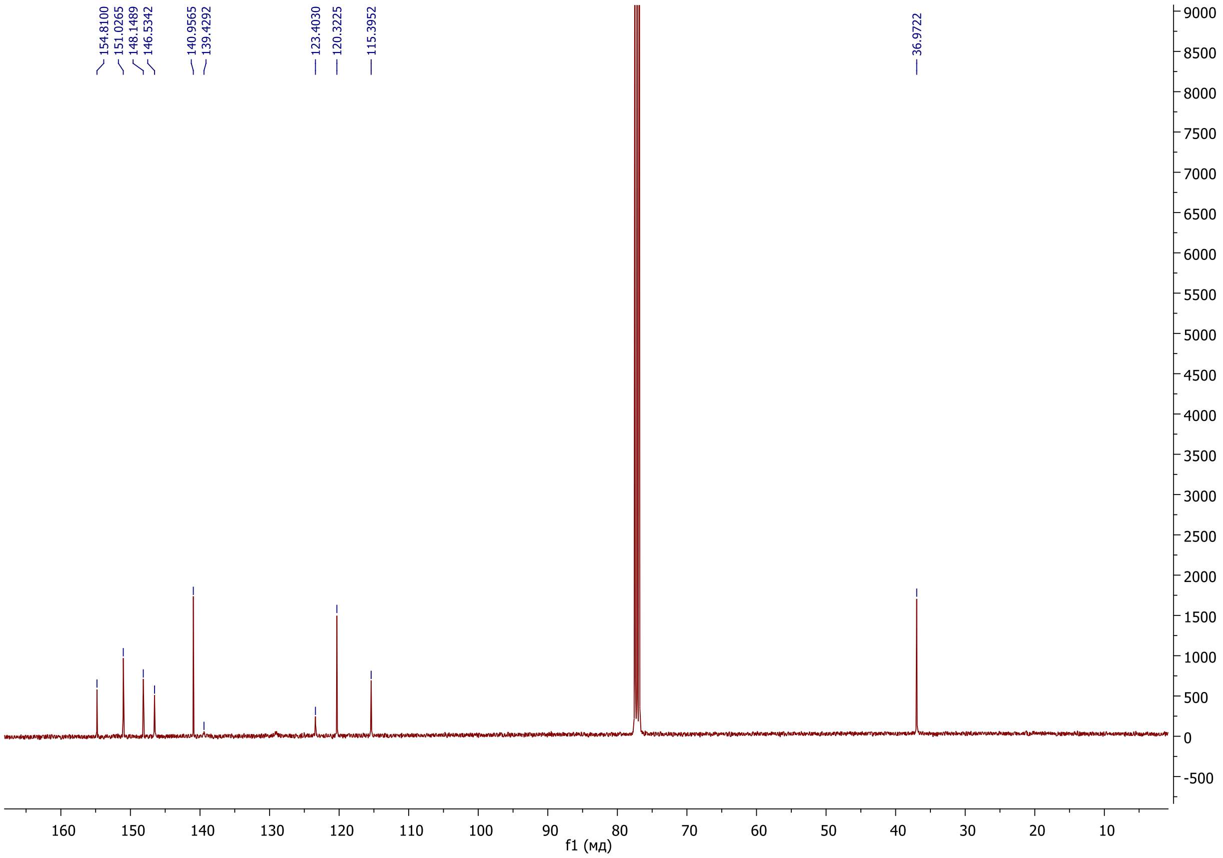

N-(5-Nitrosoisoquinolin-6-yl)pyrrolidine-1-carboxamide (10b)

NMR ^1H

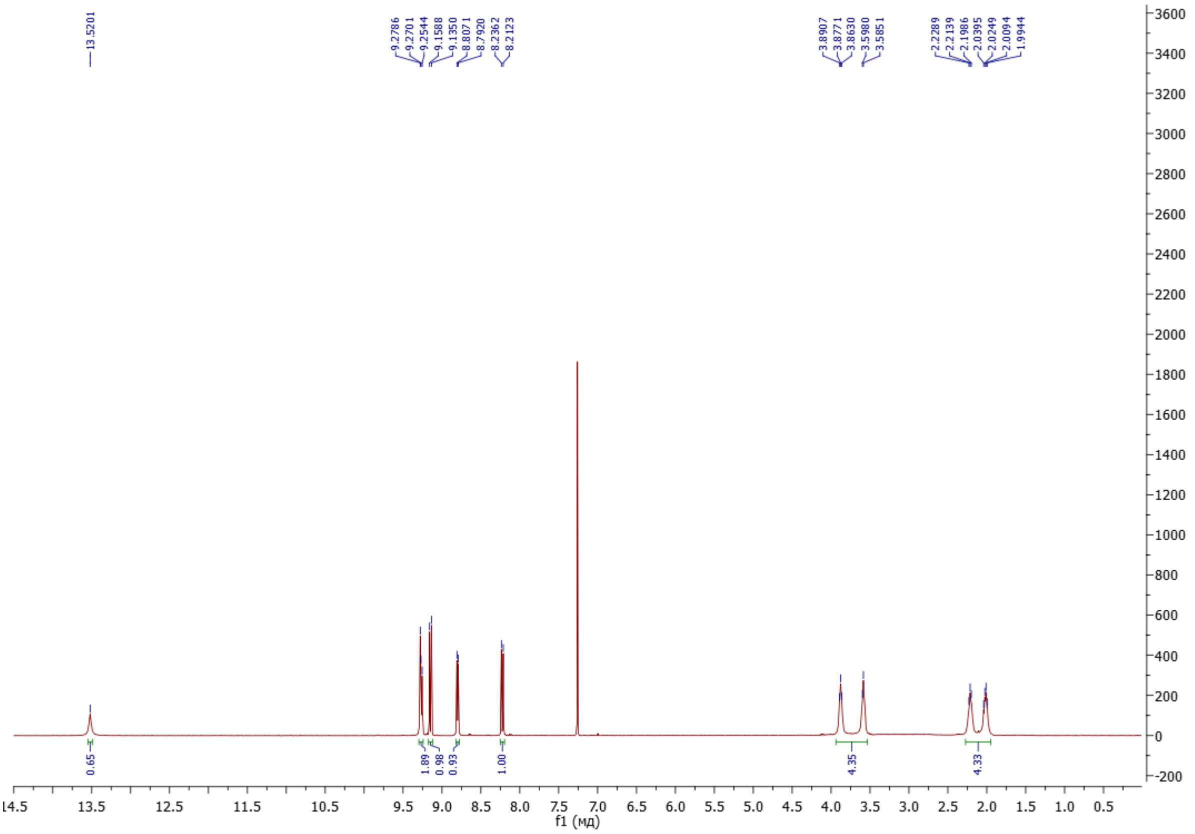

NMR ^{13}C

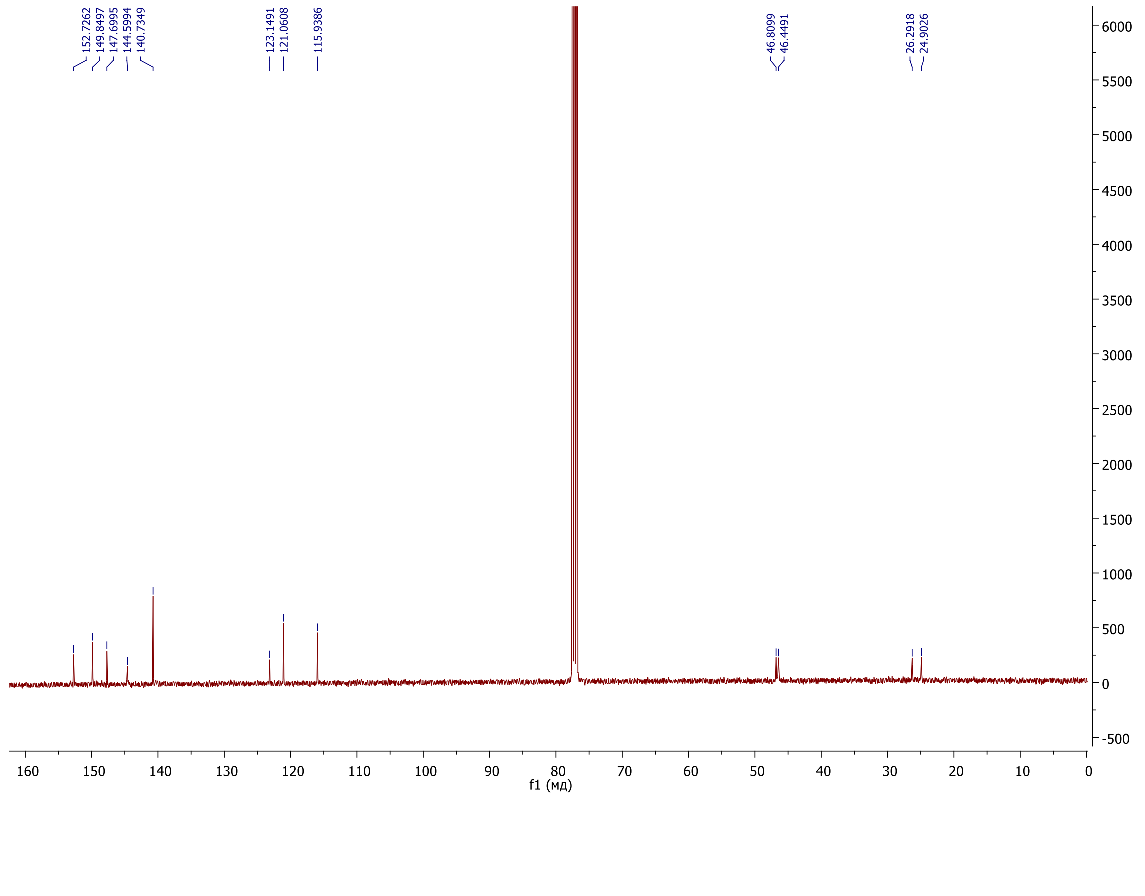

N-(5-Nitrosoisoquinolin-6-yl)piperidine-1-carboxamide (10c)

NMR ^{1}H

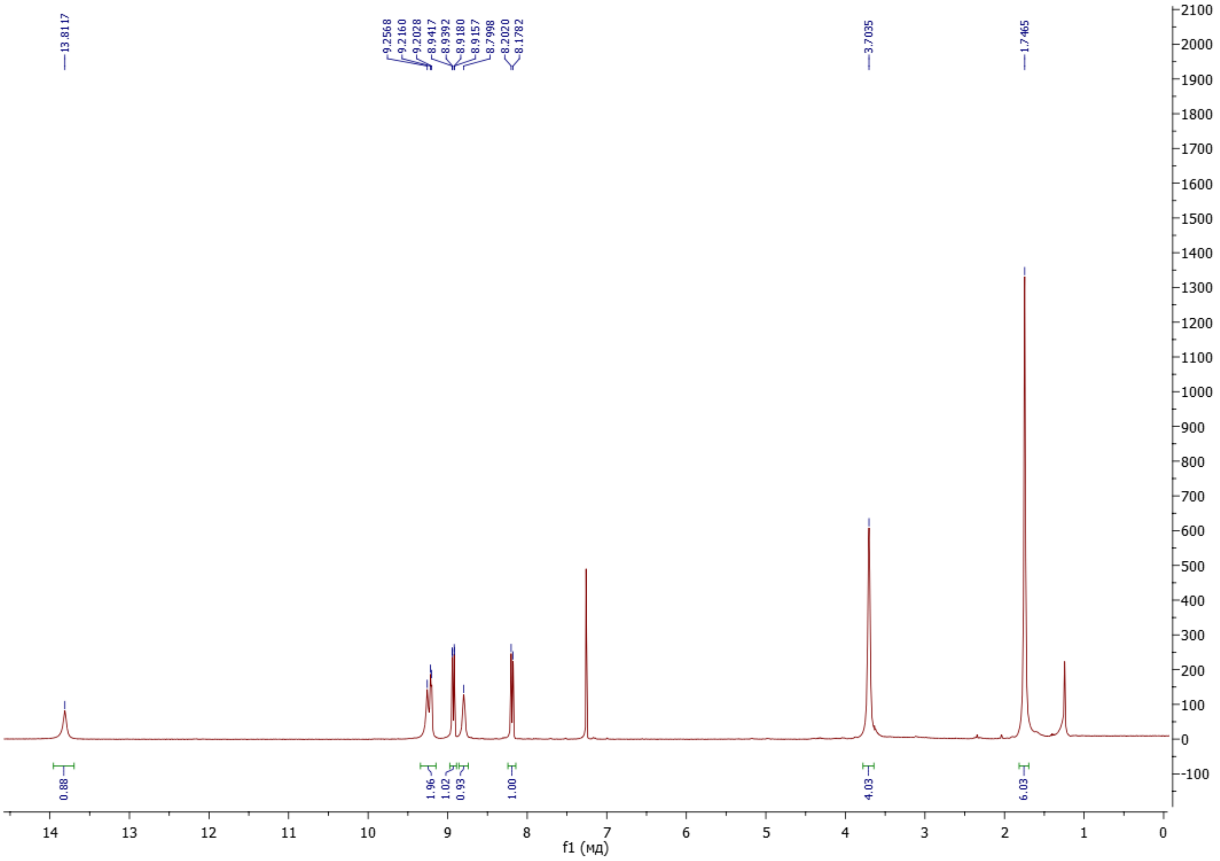

NMR ^{13}C

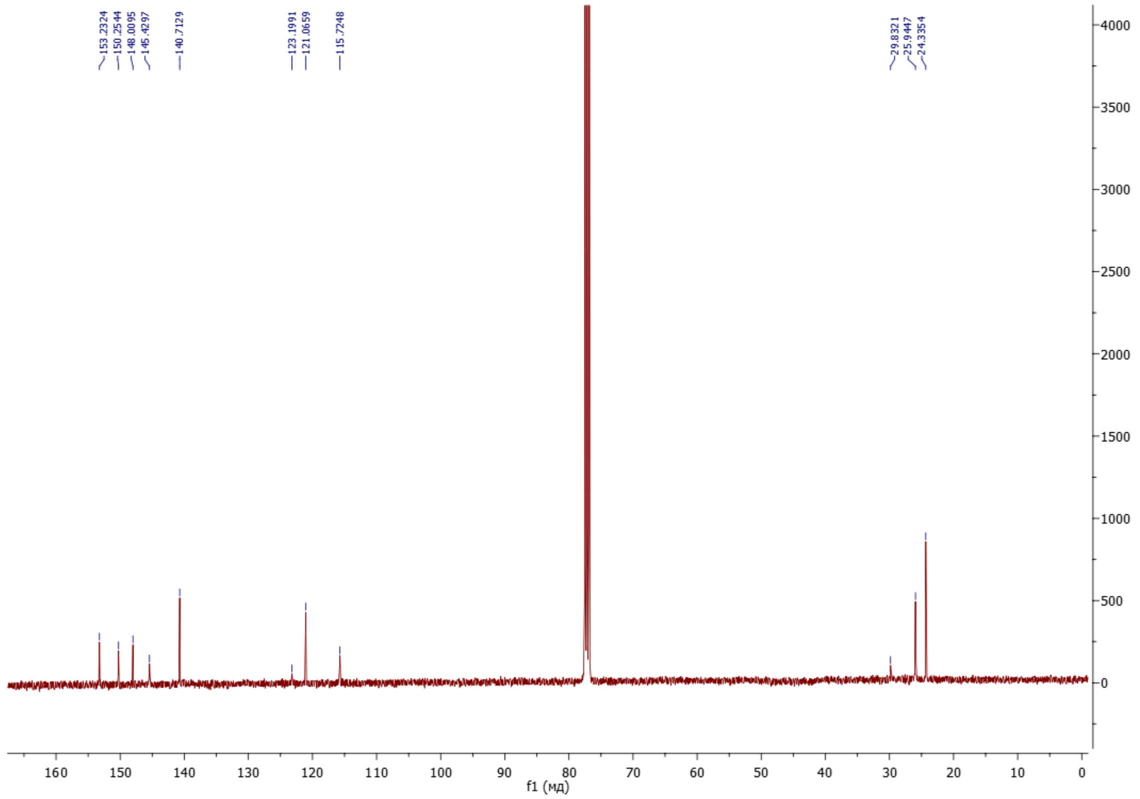

N-(5-Nitrosoisoquinolin-6-yl)morpholine-4-carboxamide (10d)

NMR ^{1}H

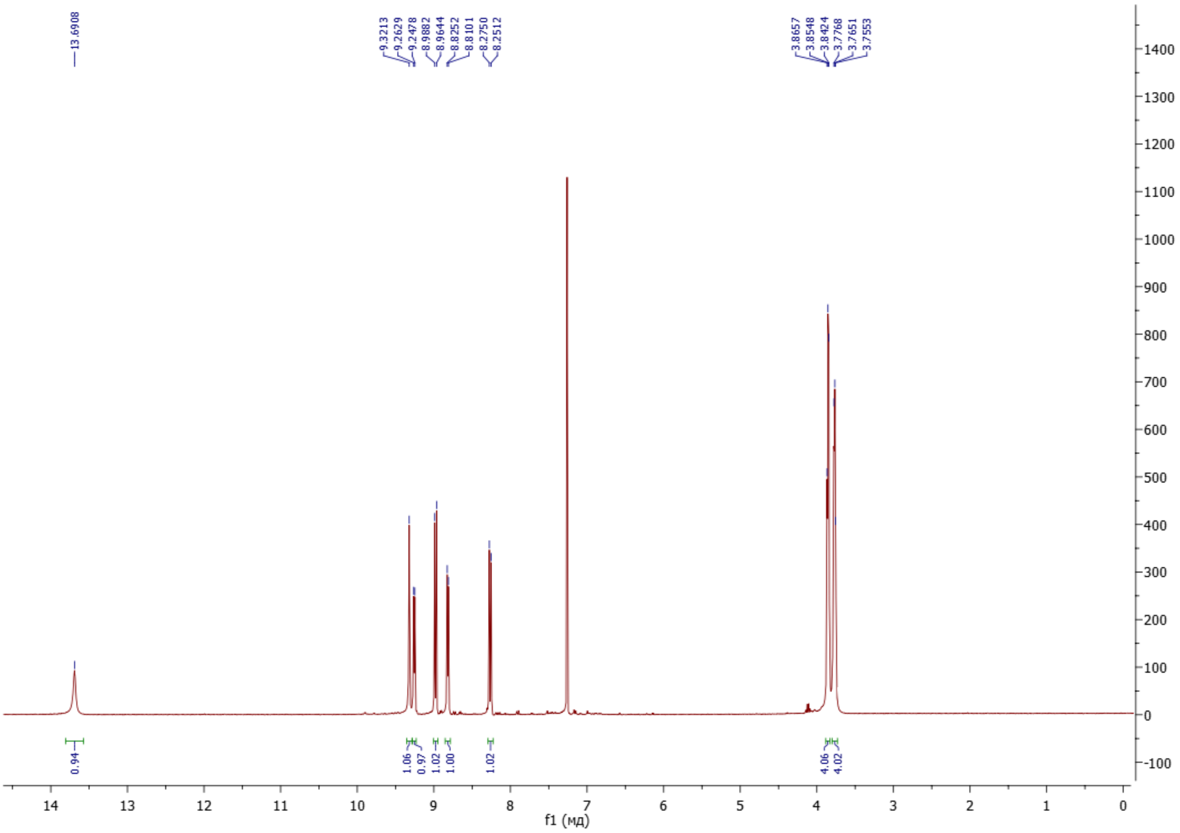

NMR ^{13}C

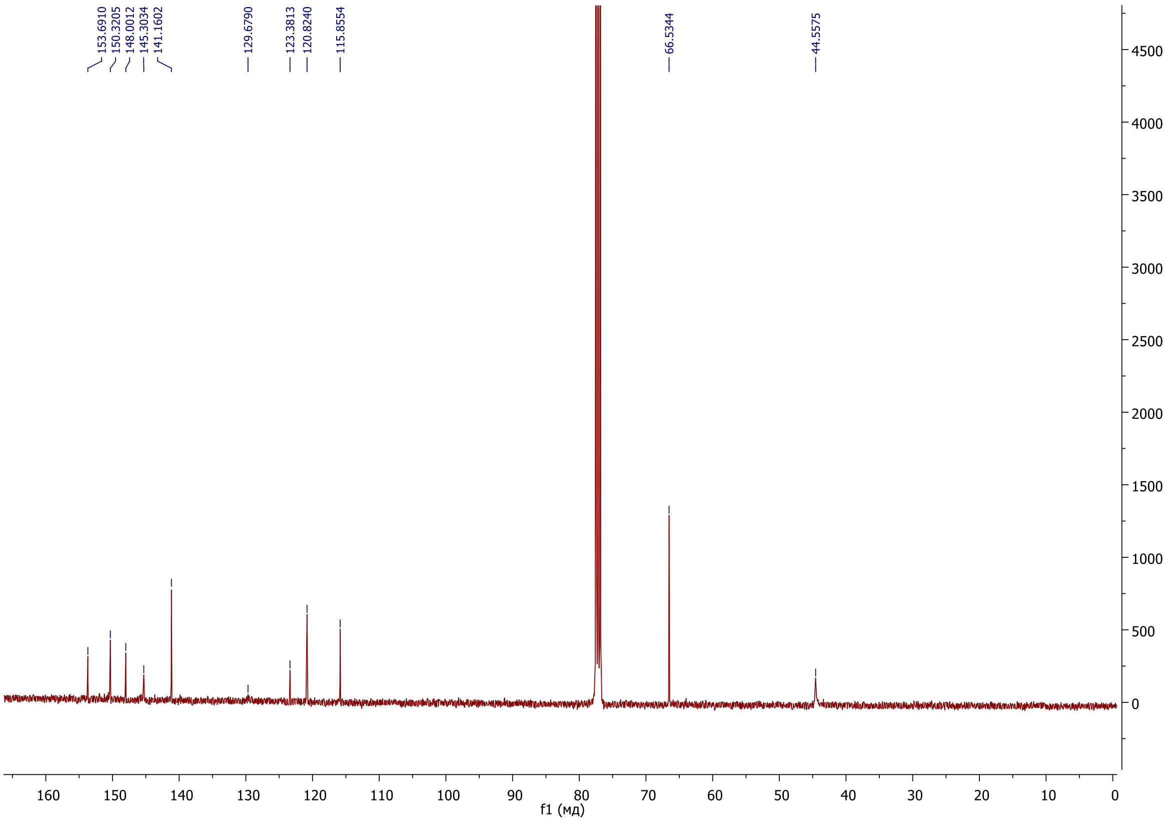

1,1-Dimethyl-3-(5-nitroisoquinolin-8-yl)urea (11a)

NMR ^{1}H

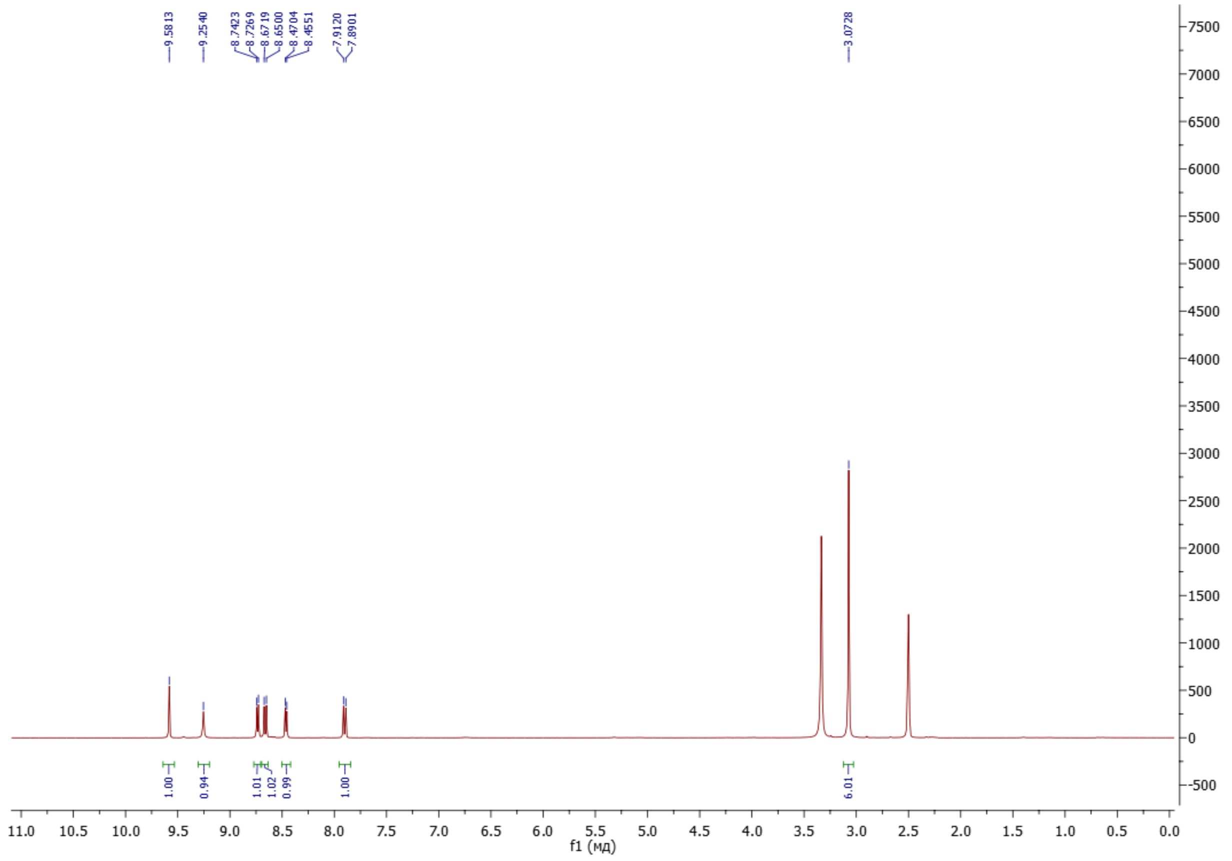

NMR ^{13}C

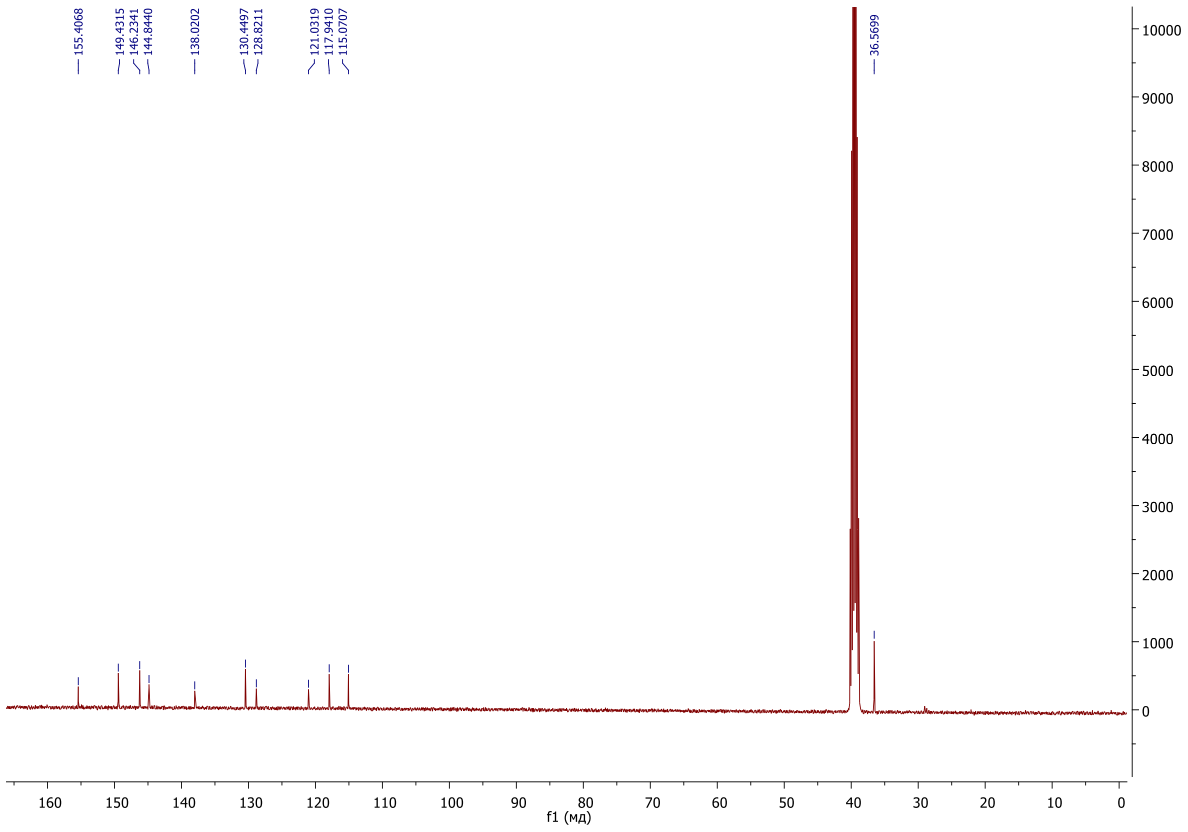

N-(5-Nitroisoquinolin-8-yl)pyrrolidine-1-carboxamide (11b)

NMR ^{1}H

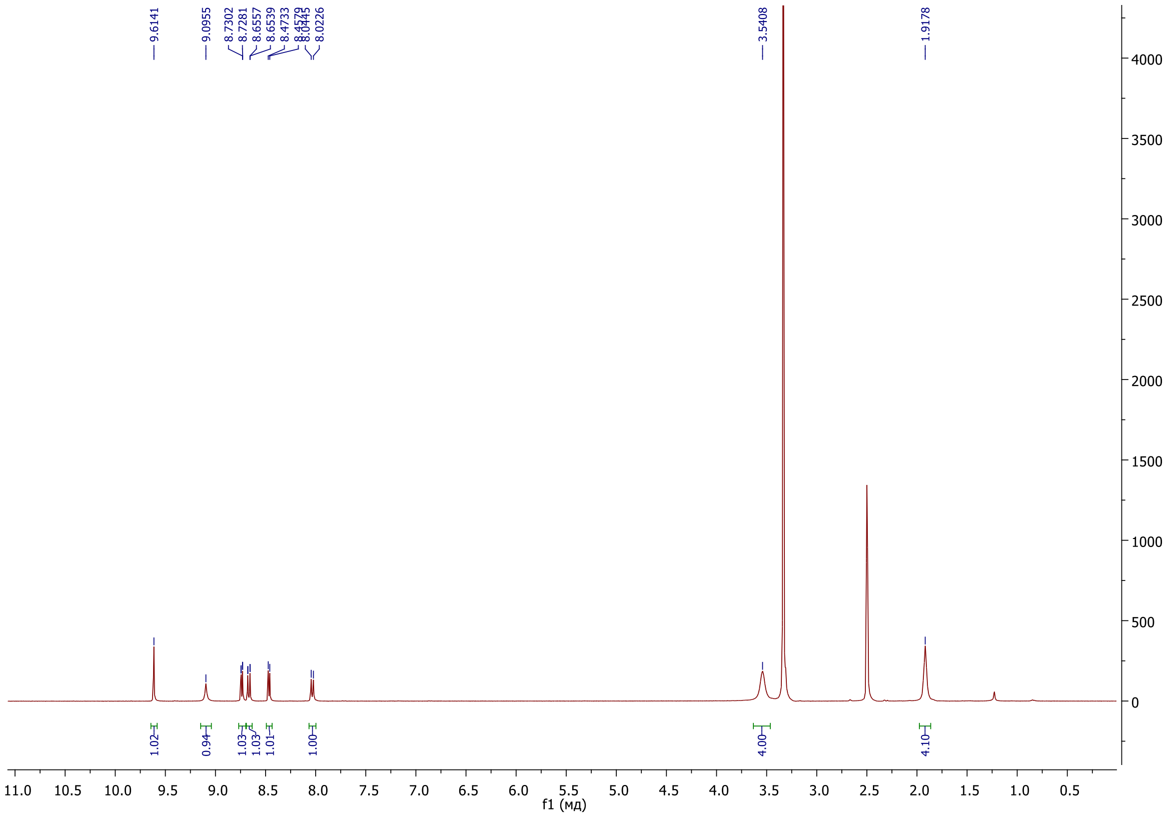

NMR ^{13}C

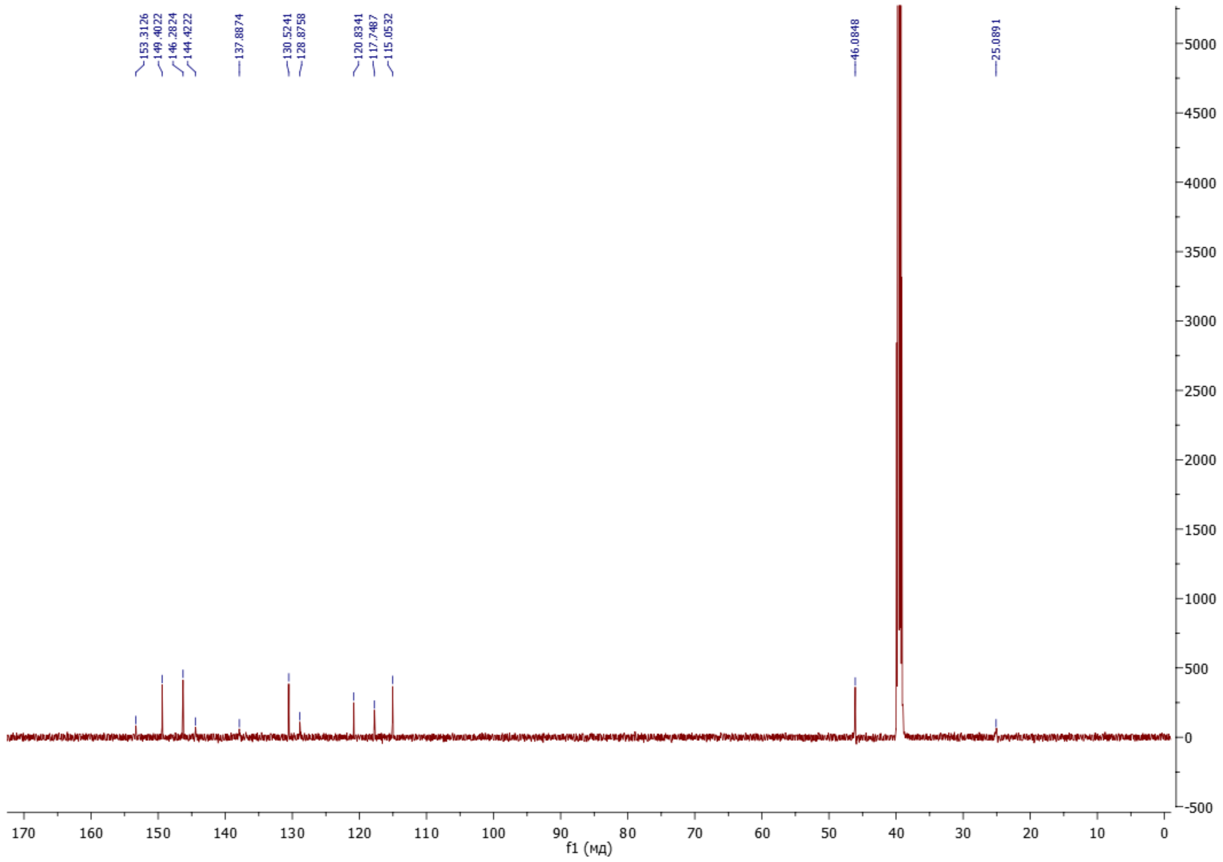

N-(5-Nitroisoquinolin-8-yl)piperidine-1-carboxamide (11c)

NMR ^{1}H

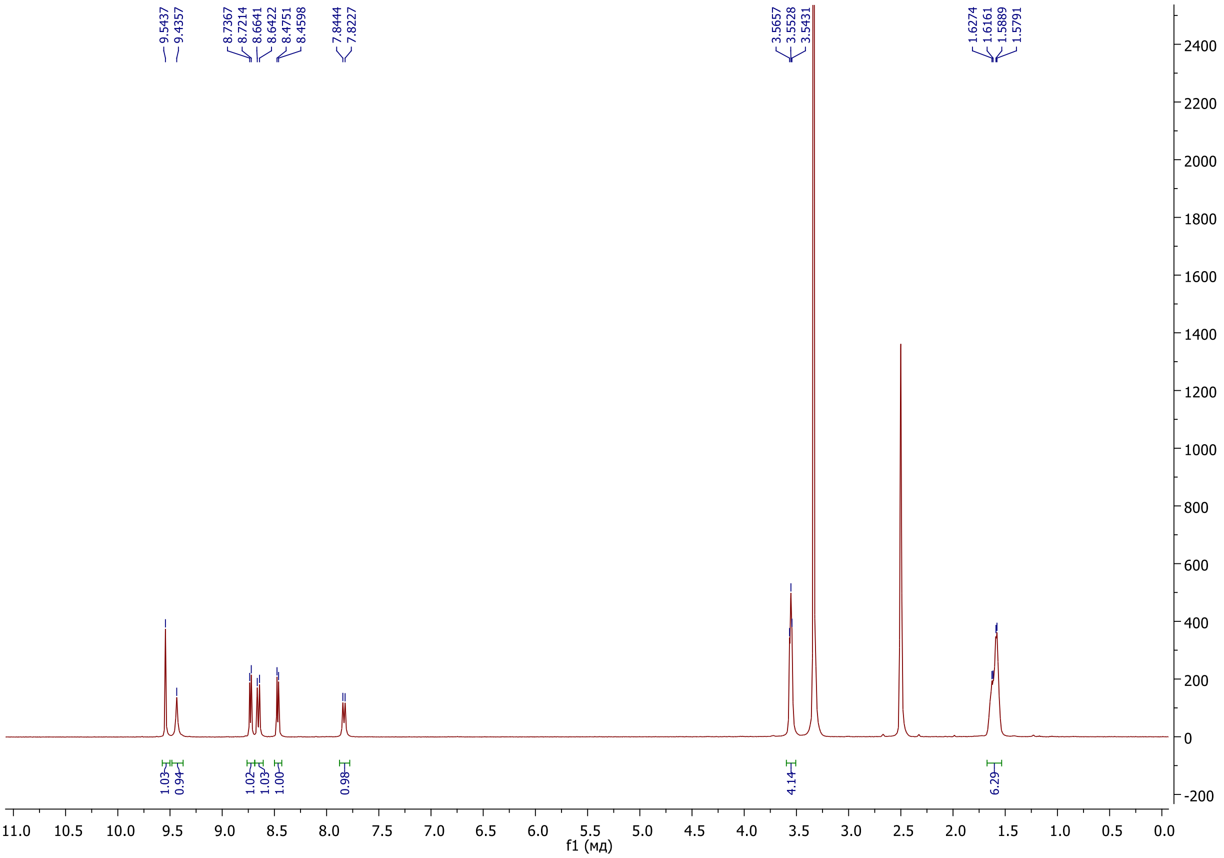

NMR ^{13}C

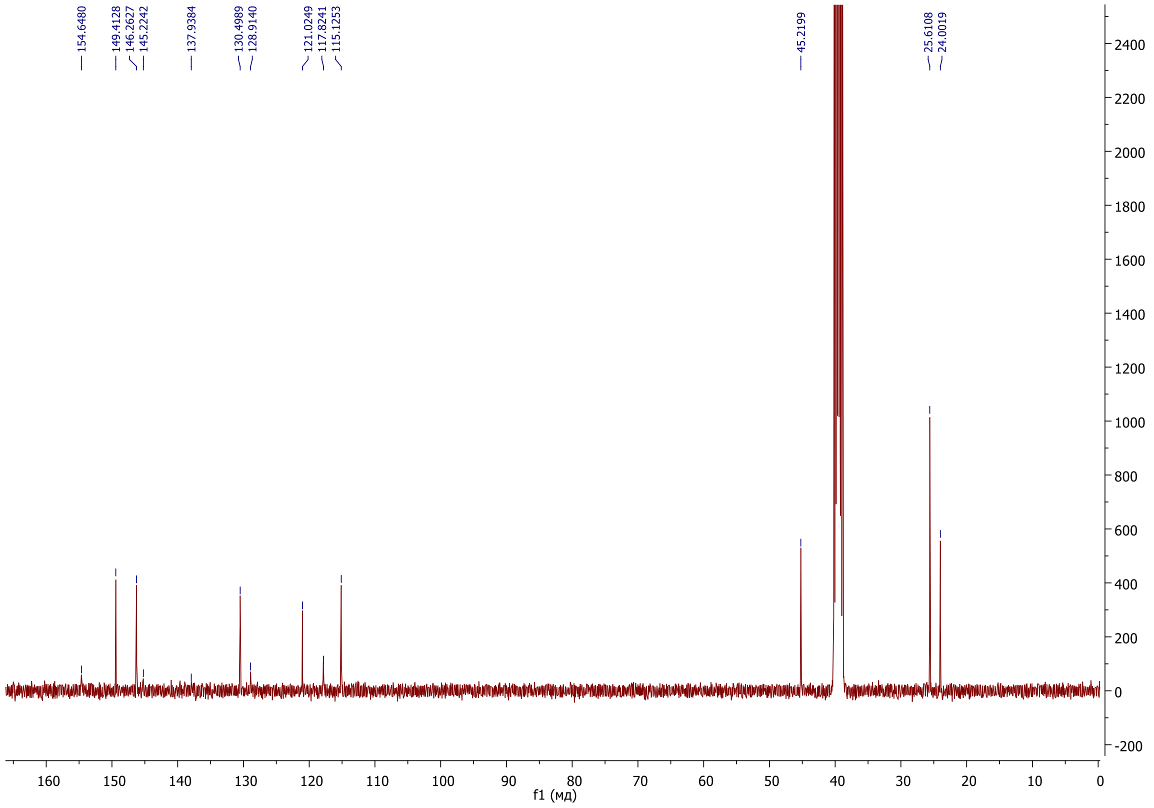

N-(5-Nitroisoquinolin-8-yl)morpholine-4-carboxamide (11d)

NMR ¹H

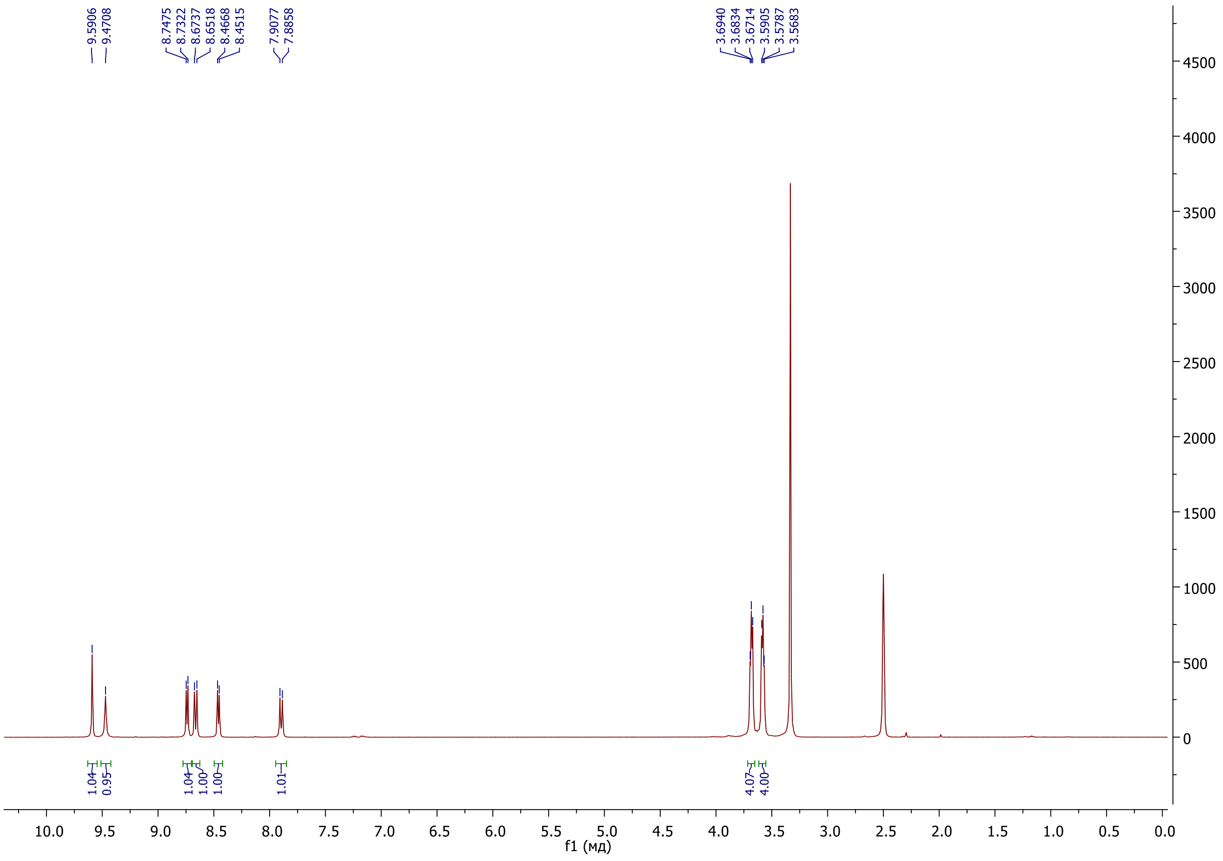

NMR ¹³C

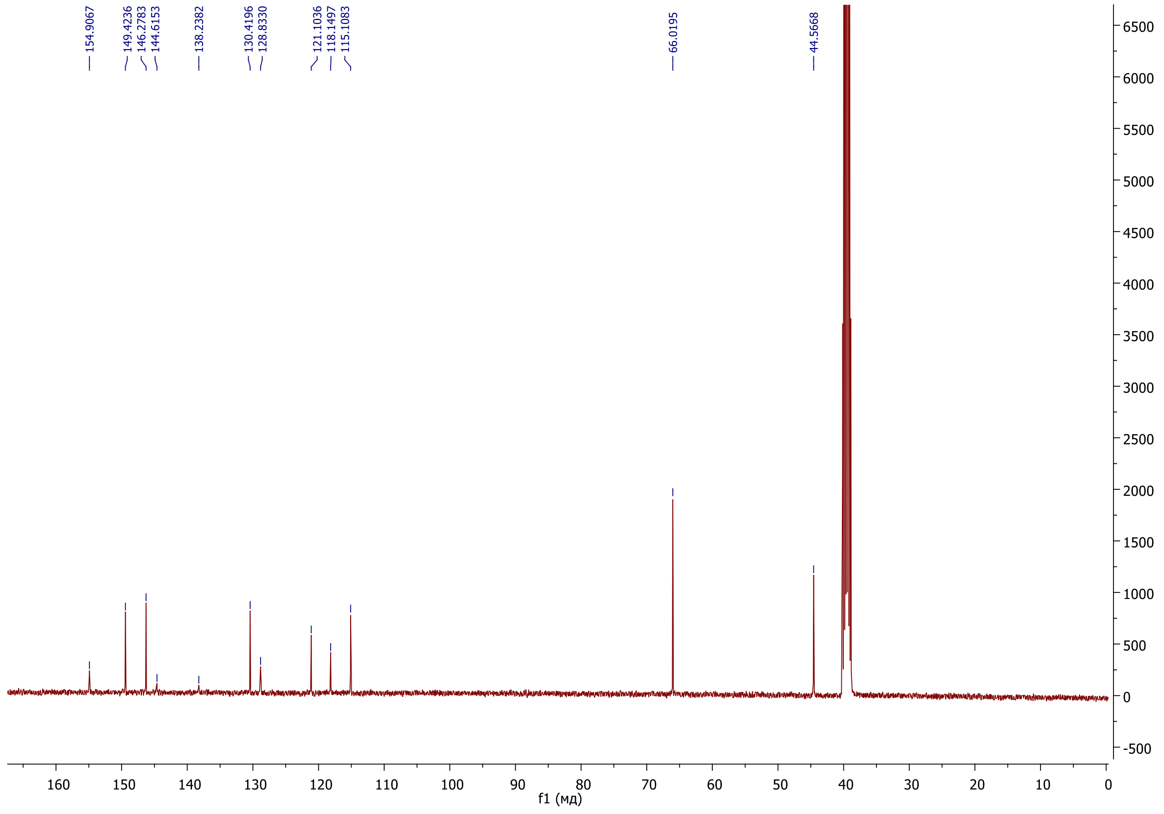

X-Ray data for the compounds 2a, 3a и 10a

Table S1 Crystal data and structure refinement for 4-methyl-*N*-(5-nitroisoquinolin-8-yl)benzamide (2a)

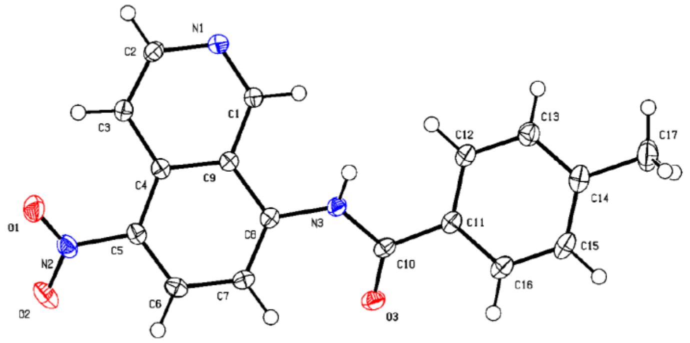

CCDC Number	2159575
Empirical formula	$C_{17}H_{13}N_3O_3$
Formula weight	307.30
Temperature/K	100.00(10)
Crystal system	monoclinic
Space group	$P2_1/c$
a/Å	14.73710(10)
b/Å	13.69850(10)
c/Å	7.11210(10)
α/°	90
β/°	96.4530(10)
γ/°	90
Volume/Å^3	1426.67(2)
Z	4
ρ_{calc}g/cm^3	1.431

μ/mm^{-1}	0.832
F(000)	640.0
Crystal size/mm^3	0.455 × 0.217 × 0.095
Radiation	CuKα (λ = 1.54184)
2Θ range for data collection/°	8.84 to 152.826
Index ranges	$-18 \leq h \leq 18$, $-16 \leq k \leq 17$, $-8 \leq l \leq 6$
Reflections collected	15246
Independent reflections	2990 [R_{int} = 0.0407, R_{sigma} = 0.0263]
Data/restraints/parameters	2990/0/213
Goodness-of-fit on F^2	1.042
Final R indexes [I>=2σ (I)]	R_1 = 0.0397, wR_2 = 0.1053
Final R indexes [all data]	R_1 = 0.0415, wR_2 = 0.1076
Largest diff. peak/hole / e Å^{-3}	0.30/-0.38

Table S 2 Crystal data and structure refinement for 4-methyl-*N*-(5-nitrosoisoquinolin-6-yl)benzamide (3a)	
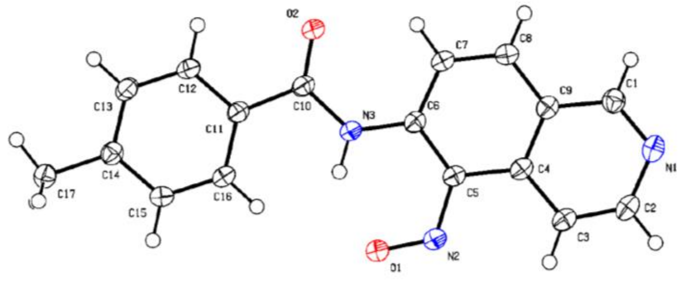	
CCDC Number	2159573
Empirical formula	$C_{17}H_{13}N_3O_2$
Formula weight	291.30

Temperature/K	100.00(10)
Crystal system	monoclinic
Space group	$P2_1/n$
a/Å	14.7376(2)
b/Å	4.70330(10)
c/Å	19.6098(3)
α/°	90
β/°	93.711(2)
γ/°	90
Volume/Å^3	1356.41(4)
Z	4
ρ_{calc}g/cm^3	1.426
μ/mm^{-1}	0.786
F(000)	608.0
Crystal size/mm^3	0.395 × 0.087 × 0.06
Radiation	CuKα (λ = 1.54184)
2Θ range for data collection/°	7.282 to 153.356
Index ranges	$-18 \le h \le 18$, $-5 \le k \le 4$, $-24 \le l \le 24$
Reflections collected	14900
Independent reflections	2822 [R_{int} = 0.0308, R_{sigma} = 0.0214]
Data/restraints/parameters	2822/0/204
Goodness-of-fit on F^2	1.065

Final R indexes [I>=2σ (I)]	R_1 = 0.0386, wR_2 = 0.1022
Final R indexes [all data]	R_1 = 0.0423, wR_2 = 0.1055
Largest diff. peak/hole / e Å^{-3}	0.28/-0.19

Table S3 Crystal data and structure refinement for 1,1-dimethyl-3-(5-nitrosoisoquinolin-6-yl)urea (10a)

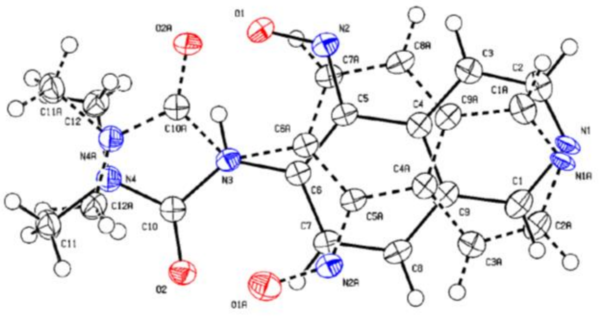

CCDC Number	2159579
Empirical formula	$C_{12}H_{12}N_4O_2$
Formula weight	244.26
Temperature/K	100.00(10)
Crystal system	triclinic
Space group	P-1
a/Å	7.0648(2)
b/Å	8.1111(3)

c/Å	10.3379(3)
α/°	89.151(3)
β/°	73.716(3)
γ/°	80.445(3)
Volume/Å^3	560.43(3)
Z	2
ρ_{calc}g/cm^3	1.447
μ/mm^{-1}	0.852
F(000)	256.0
Crystal size/mm^3	0.479 × 0.132 × 0.1
Radiation	CuKα (λ = 1.54184)
2Θ range for data collection/°	8.916 to 152.562
Index ranges	-8 ≤ h ≤ 7, -10 ≤ k ≤ 10, -12 ≤ l ≤ 13
Reflections collected	21382
Independent reflections	2331 [R_{int} = 0.0262, R_{sigma} = 0.0124]
Data/restraints/parameters	2331/0/225

Goodness-of-fit on F^2	1.077
Final R indexes [I>=2σ (I)]	R_1 = 0.0332, wR_2 = 0.0908
Final R indexes [all data]	R_1 = 0.0351, wR_2 = 0.0931
Largest diff. peak/hole / e $Å^{-3}$	0.23/-0.20
